# Differential effects of RASA3 mutations on hematopoiesis are profoundly influenced by genetic background and molecular variant

**DOI:** 10.1371/journal.pgen.1008857

**Published:** 2020-12-28

**Authors:** Raymond F. Robledo, Steven L. Ciciotte, Joel H. Graber, Yue Zhao, Amy J. Lambert, Babette Gwynn, Nathaniel J. Maki, Elena C. Brindley, Emily Hartman, Lionel Blanc, Luanne L. Peters

**Affiliations:** 1 The Jackson Laboratory, Bar Harbor, Maine, United States of America; 2 Mount Desert Island Biological Laboratory, Salisbury Cove, Maine, United States of America; 3 Feinstein Institutes for Medical Research, Manhasset, New York, United States of America; HudsonAlpha Institute for Biotechnology, UNITED STATES

## Abstract

Studies of the severely pancytopenic *scat* mouse model first demonstrated the crucial role of RASA3, a dual RAS and RAP GTPase activating protein (GAP), in hematopoiesis. RASA3 is required for survival *in utero*; germline deletion is lethal at E12.5–13.5 due to severe hemorrhage. Here, conditional deletion in hematopoietic stem and progenitor cells (HSPCs) using *Vav*-i*Cre* recapitulates the null phenotype demonstrating that RASA3 is required at the stem and progenitor level to maintain blood vessel development and integrity and effective blood production. In adults, bone marrow blood cell production and spleen stress erythropoiesis are suppressed significantly upon induction of RASA3 deficiency, leading to pancytopenia and death within two weeks. Notably, RASA3 missense mutations in two mouse models, *scat* (G125V) and *hlb381* (H794L), show dramatically different hematopoietic consequences specific to both genetic background and molecular variant. The mutation effect is mediated at least in part by differential effects on RAS and RAP activation. In addition, we show that the role of RASA3 is conserved during human terminal erythropoiesis, highlighting a potential function for the RASA3-RAS axis in disordered erythropoiesis in humans. Finally, global transcriptomic studies in *scat* suggest potential targets to ameliorate disease progression.

## Author summary

Hematopoiesis is the process by which blood cells are formed. An individual must have a normal complement of red blood cells to prevent anemia, platelets to control bleeding, and white blood cells to maintain immune functions. All blood cells are derived from hematopoietic stem cells that differentiate into progenitor cells that then develop into mature circulating cells. We studied several mouse strains carrying different mutations in the gene encoding RASA3 and human CD34+ cells, which can be induced to produce blood cells in culture. We show that RASA3 is required at the earliest stages of blood formation, the stem and progenitor cells, and that the complement of genes other than RASA3, or the genetic background, profoundly alters the overall effect on blood formation. Further, the molecular nature of the mutation in RASA3 also has a profound and independent effect on overall blood formation. One mutant mouse strain, designated *scat*, suffers cyclic anemia characterized by severe anemic crisis episodes interspersed with remissions where the anemia significantly improves. Comparison of *scat* crisis and remission hematopoietic stem and progenitor cells reveals striking differences in gene expression. Analyses of these expression differences provide clues to processes that potentially drive improvement of anemia in *scat* and provide new avenues to pursue in future studies to identify novel therapeutics for anemia.

## Introduction

RASA3 (RAS p21 protein activator 3), also called GAPIII or IP4BP (inositol 1,3,4,5-tetrakisphosphate, IP4, binding protein), is a member of the GAP1 family of RAS-GTPase-activating proteins (GAPs) that also includes RASA2 (GAP1^m^), RASA4 (CAPRI) and RASAL1 (RASAL)[1]. Small GTPases act as molecular switches, cycling between active GTP-bound and inactive GDP-bound forms. They are activated by guanine nucleotide exchange factors (GEFs), which stimulate GTP loading, and inactivated by GAPs, which accelerate GTP hydrolysis. Membrane localization is required for GAP activity for all GAP1 family members, which are highly conserved and share a common structure consisting of dual N-terminal C2 domains, a central catalytic GAP domain, and C-terminal pleckstrin homology (PH) and Bruton tyrosine kinase (Btk) domains [2–4].

With the exception of RASA2, members of the GAP1 subfamily are dual RAS and RAP GAPs [4–6]. RAS is a critical mediator of cytokine-dependent signaling in erythropoiesis (erythropoietin, Epo; stem cell factor, SCF)[7–9] and thrombopoiesis (thrombopoietin, TPO)[10], transmitting signals to multiple effector pathways that regulate cell proliferation, differentiation, survival, adhesion, and actin cytoskeleton organization. RAP proteins are highly expressed in endothelial cells, leukocytes, and platelets and play major roles in cell polarity, adhesion, and movement [11].

Defects in adhesion, as reflected by severe hemorrhage and reduced numbers of adherens junctions between endothelial cells in vessels of the brain, were noted in the first knockout of *Rasa3* in mice where exons 11 and 12 within the central catalytic GAP domain were replaced with a neomycin cassette; the model was embryonic lethal at E12.5–13.5 [12]. A major role for RASA3 in hematopoiesis was first identified upon positional cloning of the co-isogenic autosomal recessive mouse mutation, *scat* (severe combined anemia and thrombocytopenia) [13]. The *scat* phenotype, in addition to severe anemia and thrombocytopenia, includes significant leukopenia as well. The *scat* disease progresses episodically, with periods of severe crisis interspersed with one or two periods of remission. Early studies showed that the *scat* disease phenotype, including its episodic nature, is fully transferrable to wild type mice via hematopoietic stem cell transplantation using *scat* bone marrow or spleen-derived stem cells [14]. Notably, full remission as presented in the original description [14] of the *scat* disease, is now exceedingly rare. Instead, partial remission occurs, which is restricted to the erythroid component–leukocyte and platelet numbers do not recover. This change in *scat* disease progression is likely due to genetic drift over the thirty years since its original description, admixture of two BALBc/By sublines (see Methods), and environmental changes (e. g., changes in animal room health status standards established and maintained since the late 1980s). The RASA3 mutation (G125V) in *scat* causes mislocalization of RASA3 to the cytosol, abrogating RASA3 GAP activity and increasing active RAS levels in *scat* erythroid cells. Increased active RAS is associated with delayed terminal erythroid differentiation [8,9]. Indeed, terminal erythropoiesis in *scat* is significantly delayed at the poly- and orthochromatophilic developmental stages [13].

Additional studies in mouse models have defined RAP-dependent RASA3 functions *in vivo*. Studies in *hlb381*, a recessive chemically (N-Ethyl-N-Nitrosourea, ENU) induced model carrying a different RASA3 missense mutation (H794L), confirmed RASA3 as a critical inhibitor of RAP1-dependent integrin signaling and platelet activation [15]. Studies in conditional catalytically inactive RASA3 models as well as models created by transplant of *Rasa3* KO E12.5 fetal liver cells to wild type (WT) hosts revealed RAP1-dependent defects in megakaryocyte integrin signaling and structure (disorganized actin cytoskeleton), platelet adhesion and activation, endothelial cell adhesion and vascular lumen integrity [16,17].

Here, we sought to further analyze the role of RASA3 in hematopoiesis using conditional knockout models to determine the status of HSPCs in RASA3 deficiency, to elucidate mechanistic differences underlying H794L (*hlb381*) and G125V (*scat*), to perform RNAseq studies to generate novel hypotheses regarding the progression of the *scat* disease from periods of crisis (cr) to partial remission (pr), and to assess the potential role of RASA3 in human erythropoiesis. Our results show that (1) there is a strict requirement for RASA3 during development and into adulthood at the level of HSPCs in order for hematopoiesis to progress normally, (2) adult bone marrow hematopoiesis and stress erythropoiesis in the spleen are suppressed upon induction of RASA3 deficiency, (3) genetic background and the nature of the molecular variant profoundly influence the RASA3 deficient phenotype, (4) global transcriptomics reveals massive expression differences in *scat vs*. WT hematopoietic tissues and cells, generating novel hypotheses on potential mechanisms leading to spontaneous disease amelioration (partial remission), and (5) the role of RASA3 is conserved during human terminal erythropoiesis.

## Results

### Deletion of *Rasa3* in erythroid progenitors and precursors leads to normal erythropoiesis

**Fig 1:**
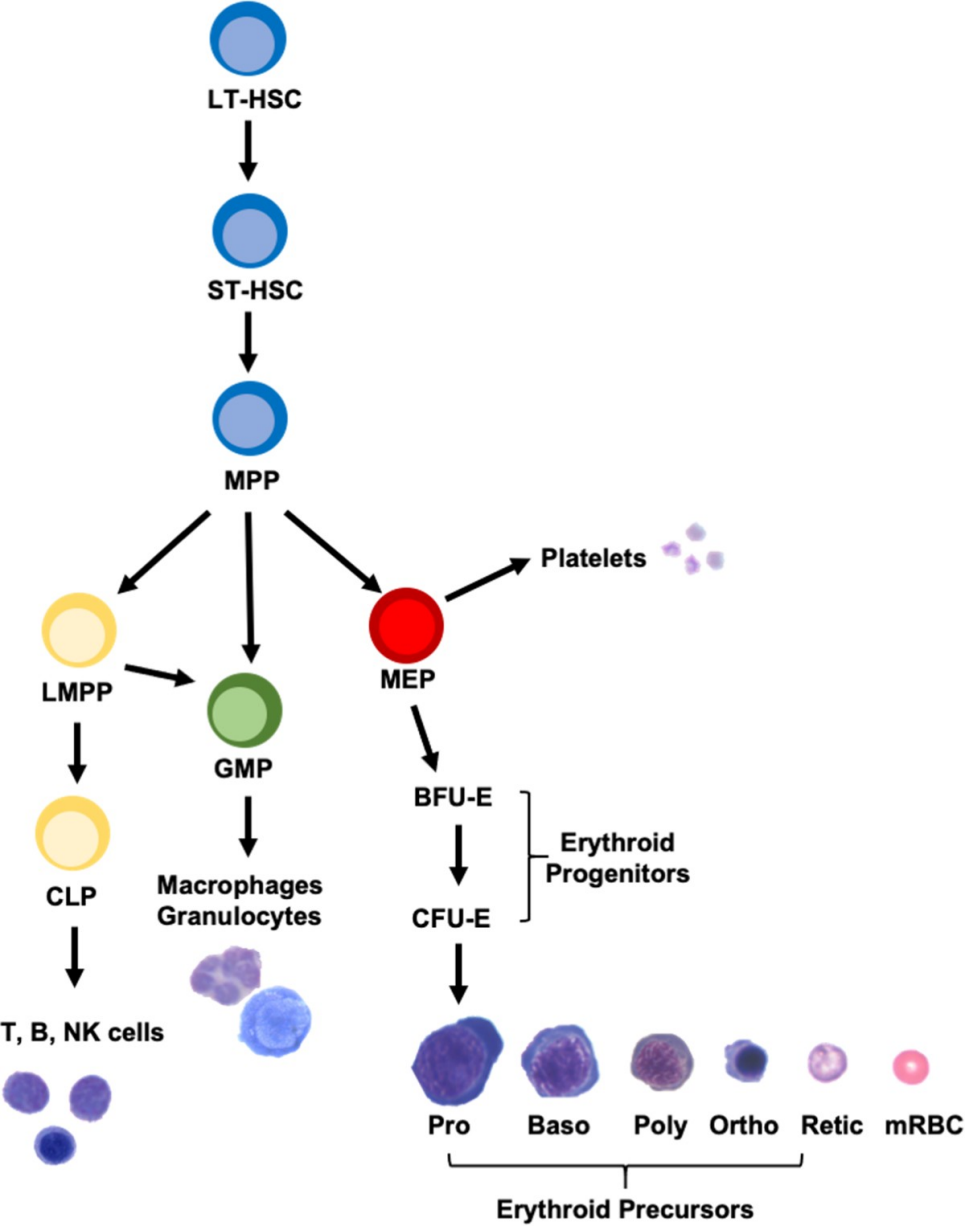
Schematic showing development of hematopoietic stem cells into mature blood cells. The experimental evidence indicates that RASA3 is required throughout developmental hematopoiesis beginning at the HSPC stage. See text for details. LT-HSC, long term hematopoietic stem cell; ST-HSC, short term hematopoietic stem cell; MPP, multipotent progenitor; LMPP, lymphoid-primed multipotent progenitor; CLP, common lymphoid progenitor; GMP, granulocyte monocyte progenitor; MEP, megakaryocyte-erythroid progenitor, BFU-E, burst forming unit-erythroid; CFU-E, colony forming unit-erythroid; Pro, proerythroblast; Baso, basophilic erythroblast; Poly, polychromatic erythroblast; Ortho, orthochromatic erythroblast; Retic, reticulocyte; mRBC, mature red blood cell.

**Fig 2:**
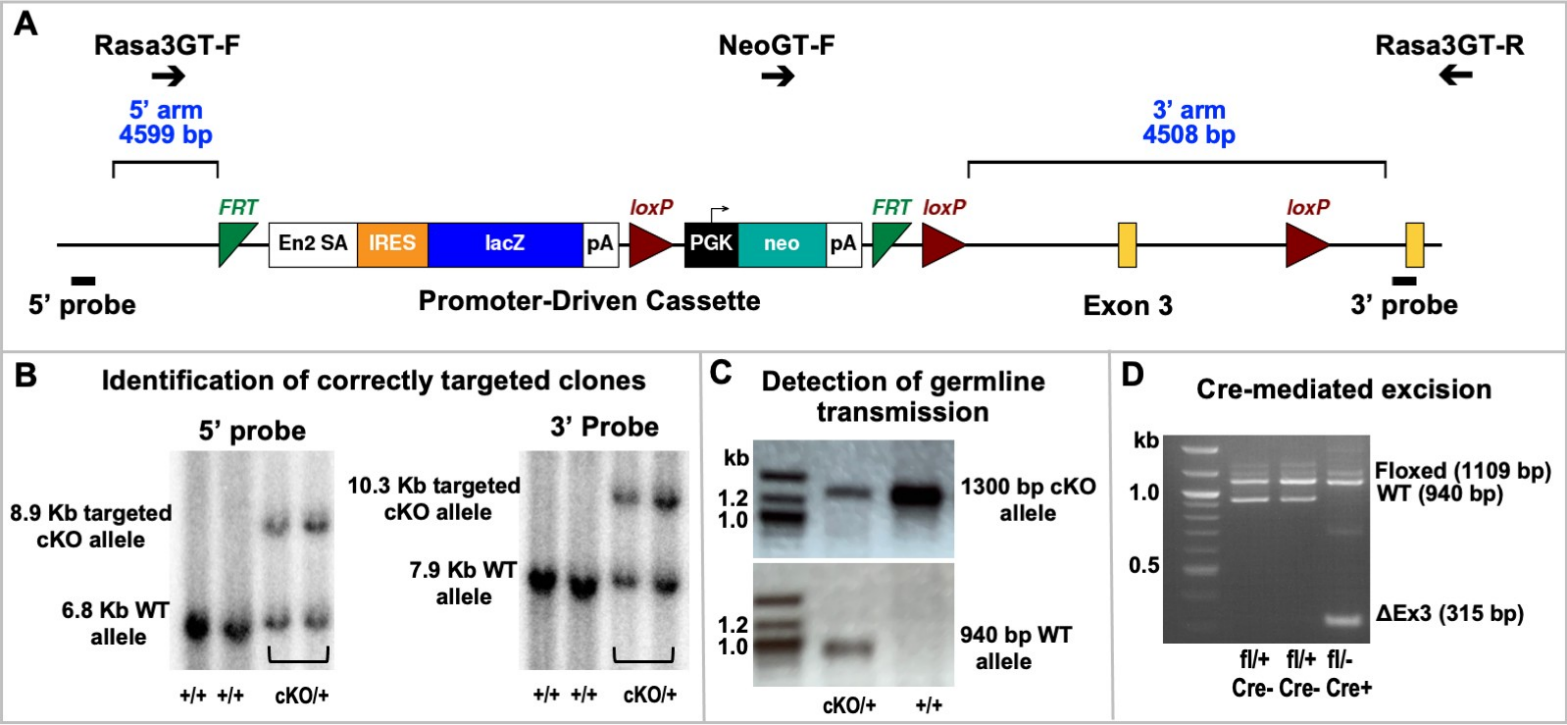
Generation of *Rasa3* conditional knockout (cKO) mice. **(A)** *Rasa3*^tm1a(KOMP)Wtsi^ knockout-first, promoter-driven conditional ready construct. Positions of genotyping (GT) primers, *FRT* (green) and *loxP* (red) sites, *Rasa3* exons (yellow), homology arms (blue) and flanking Southern blot probes (black rectangles) are shown. **(B)** Southern blots identifying correctly targeted cKO and wild type (WT) alleles in *BclI*- and *Bgl1*-digested ES cell genomic DNA using 5’ (left, *Bcl1* digest) and 3’ (right, *Bgl1* digest) flanking probes. **(C)** Detection of germline transmission using PCR to detect targeted cKO and WT alleles in genomic DNA. **(D)** PCR detection of *Rasa3* alleles following Flpe-mediated recombination to delete the promoter-driven cassette and Cre-mediated recombination to delete exon 3. Primer sequences for PCR are provided in S10 Table.

Blood formation begins with pluripotent hematopoietic stem cells (HSCs) that both self-renew and differentiate into various hematopoietic progenitor cells (HPCs) that become progressively restricted in their developmental potential but, collectively, produce all the blood cell lineages in the circulation (Fig 1). Erythroid progenitors differentiate through four morphologically identifiable erythroid precursor stages (pro-, basophilic, polychromatophilic, and orthochromatic erythroblasts) during terminal erythroid differentiation to form reticulocytes and, finally, mature red cells (Fig 1). Our previous work demonstrated that *Rasa3* plays a critical role in erythropoiesis. In *scat*, we observed an accumulation of polychromatophilic and orthochromatic erythroblasts both in the bone marrow and in the spleen during crisis episodes. To understand the mechanism(s) leading to this accumulation in late precursor stages, we generated conditional knockout (cKO) alleles of *Rasa3* as detailed in Methods and summarized in Fig 2. We first generated *Rasa3*^*fl*/-^ and *Rasa3*^*fl/fl*^ mice carrying *Epor-Cre* (S1 Fig). *Cre* expression is driven by the erythropoietin receptor promoter beginning at the BFU-E stage and continuing through the proerythroblast stage of terminal erythroid differentiation [18]. Surprisingly, all *Epor-Cre; Rasa3* mutant offspring were normal in appearance at birth and at 6–8 weeks of age despite efficient deletion of exon 3 in red cell precursors (S2A Fig). Complete blood counts in adults did not differ from control in any parameters except for slightly decreased platelet counts and increased circulating reticulocytes (S1 Table), neither of which reached clinically significant levels nor approached the magnitude of changes seen in *scat* homozygotes (S2 Table). Spleen weight and peripheral blood morphology were normal as well in *Epor-Cre; Rasa3* mutant mice (S2B and S2C Fig). Western blotting of red cell membrane ghosts confirmed that abundant RASA3 protein was present in mutant red cell membranes (S2D Fig). Together, these data suggest that RASA3 produced prior to the BFU-E stage of erythropoiesis persists during terminal erythroid differentiation and is sufficient to sustain normal erythropoiesis.

### Deletion of *Rasa3* in hematopoietic stem and progenitor cells (HSPCs) and endothelial cells (ECs) recapitulates the null phenotype

**Fig 3:**
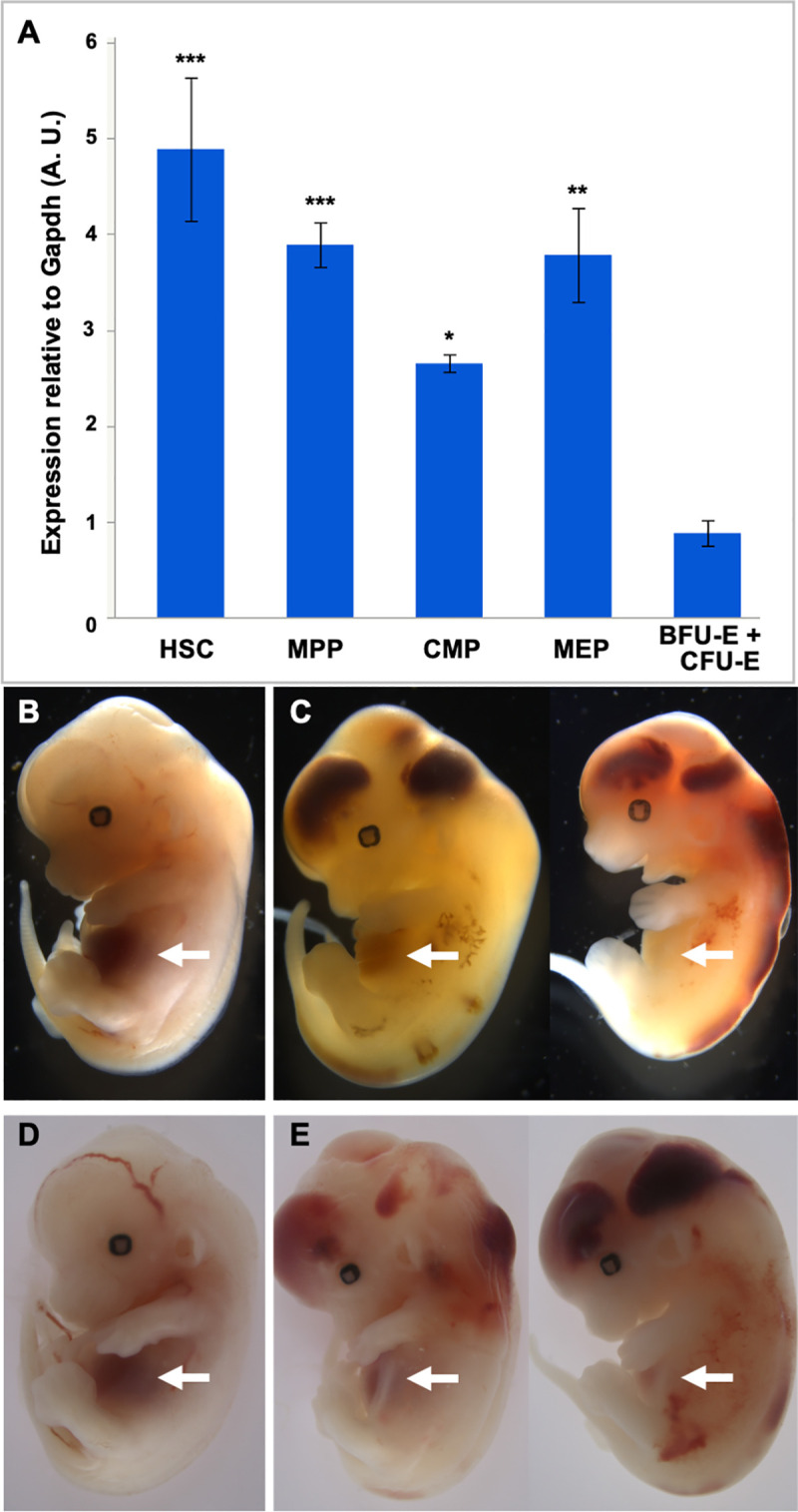
Deletion of *Rasa3* in hematopoietic and endothelial cells. **(A)** Expression of *Rasa3* in flow sorted adult B6J HSPCs (X ± SEM, n = 3 pools of cells from 6 mice for all cell types). **(B)** E12.5-E13.5 *Vav-Cre*; *Rasa3* control and **(C)** mutant embryos. **(D)** E12.5-E13.5 *Tek-Cre; Rasa3* control and mutant **(E)** embryos. Mutant embryos recapitulate the germline null phenotype with severe hemorrhage and decreased fetal liver erythropoiesis (arrows). HSC, hematopoietic stem cells; MPP, multipotent progenitors; CMP, common myeloid progenitor; MEP, Megakaryocyte-erythroid progenitor; BFU-E, burst forming unit-erythroid; CFU-E, colony forming unit-erythroid. *p < 0.05, **p < 0.01, ***p < 0.001 *vs*. BFU-E/CFU-E.

To test the hypothesis that sufficient stable RASA3 protein is produced prior to the BFU-E stage to maintain normal terminal erythroid differentiation in *Epor-Cre; Rasa3* mutant mice, we first examined *Rasa3* expression in normal C57BL/6J (B6J) HSPCs. Expression of *Rasa3*, highest in early HSCs, declines by more than 80% in an enriched mixture [19] of BFU-E and CFU-E erythroid progenitors (Fig 3A). Examination of expression data available online (nanexpression.mdibl.com [20], https://www.cbil.upenn.edu/ErythronDB/, https://kleintools.hms.harvard.edu/paper_websites/tusi_et_al/index.html [21]) reveals that *Rasa3* expression continues to decline as terminal differentiation proceeds both during primitive and definitive erythropoiesis. To delete *Rasa3* in HSPCs and ECs during development, we utilized *Vav-iCre* [22,23]. No viable *Vav-iCre; Rasa3* mutant mice were recovered at birth; all died *in utero* at E12.5–13.5 with severe hemorrhage and significantly decreased fetal liver erythropoiesis (Fig 3B and 3C), as was also observed in germline null *Sox2-Cre; Rasa3* mutants (S3 Fig). We obtained the same result when *Cre* expression was driven by Tie2 (*Tek-Cre*, Fig 3D and 3E), which is also expressed in both ECs and HSCs [23,24]. These data suggest that *Rasa3* is essential for hematopoiesis during development at the hematopoietic stem and progenitor cell level.

### Induced deletion of *Rasa3* in adults leads to severe anemia, leukopenia, and thrombocytopenia

**Fig 4:**
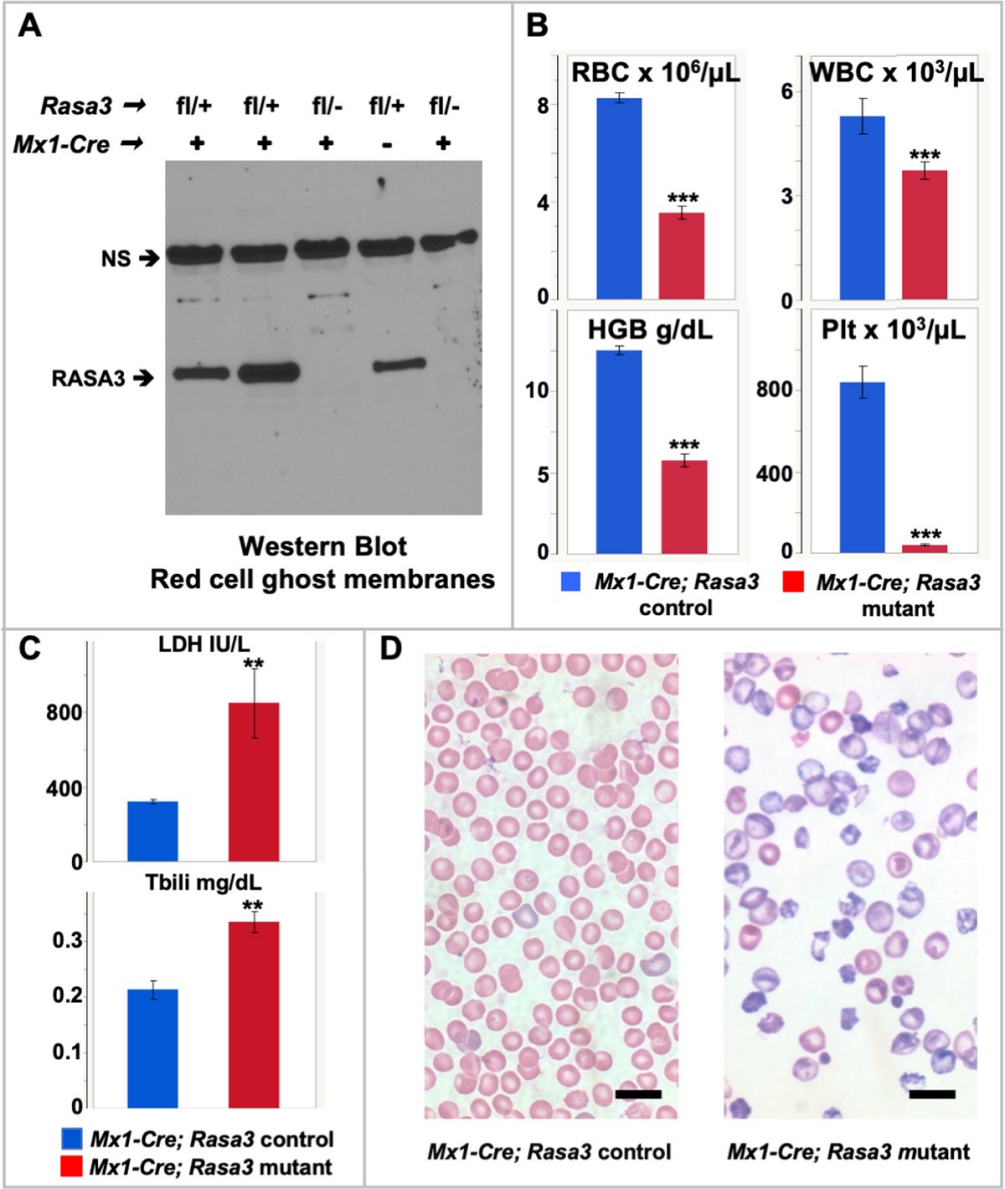
Deletion of *Rasa3* in adults leads to profound anemia. **(A)** Western blotting confirms loss of RASA3 protein in mutant red cell ghost membranes from pIpC treated *Mx1-Cre; Rasa3* adult mice. NS = non-specific band used as loading control. **(B)** Profound anemia and thrombocytopenia with significant leukopenia in pIpC treated *Mx1-Cre; Rasa3* mutant adults with **(C)** increased serum lactate dehydrogenase (LDH) and total bilirubin (Tbili) and **(D)** strikingly abnormal peripheral blood morphology, which arises from hemolysis as well as stress erythropoiesis itself. Hemolysis is known to cause misshapen red cells. In stress erythropoiesis, reticulocytes are released from the bone marrow and spleen to the circulation earlier than normal. Early, immature reticulocytes are large, multilobulated, and full of RNA and organelles. RNA results in a dark gray-purple appearance on Wright’s stained smears. Reticulocyte maturation in the circulation includes extensive membrane remodeling that leads to loss of surface area and elimination of organelles, producing the mature and smaller erythrocyte. Values in B and C, X ± SEM for males only; complete CBC data is given for both sexes in Table 1. C, n = 4 (control) and 6 (mutant). Bar, 10 μM. *p < 0.05, **p < 0.01, ***p < 0.001.

Having established a critical role for *Rasa3* in blood formation during development, we next utilized *Mx1-Cre* transgenic mice to delete RASA3 throughout the hematopoietic system in adult mice upon induction with polyinosinic:polycytidylic acid (Poly(I:C))[25,26]. Western blotting confirmed loss of RASA3 protein in *Mx1-Cre; Rasa3* mutant red cell membranes within 2 weeks of the final Poly(I:C) injection (Fig 4A) at which time the mice are profoundly anemic and thrombocytopenic with significant leukopenia (Fig 4B and Table 1). Spleen weight, percent circulating reticulocytes, mean corpuscular volume, and red cell and hemoglobin distribution widths are significantly increased in *Mx1-Cre; Rasa3* mutant mice compared to controls (Table 1). Serum LDH and total bilirubin, indicative of hemolysis, are increased (Fig 4C). In previous studies [27], mechanisms known to contribute to red cell hemolysis have been documented in *scat*, including generation of excess reactive oxygen species that leads to oxidative damage to red cell membranes and, in turn, hemolysis. The peripheral blood morphology is markedly abnormal (Fig 4D). *Mx1-Cre; Rasa3* heterozygotes did not differ from control in any of these parameters (Table 1). Together, our complementary studies using *Vav*-, *Tie2*- and *Mx1-Cre* highlight that production of RASA3 in HSPCs is required to maintain terminal erythroid differentiation and to generate normal numbers of circulating mature erythroid cells as well as leukocytes and platelets.

**Table 1:** Complete blood counts in *Mx1-Cre; Rasa3* adult mice 6–8 weeks of age.

Group (n)	WBC (x10^3^/μL)	RBC (x10^6^/μL)	Hgb (g/dL)	Hct (%)	MCV (fL)	MCH (pg)	MCHC (g/dL)	RDW (%)		HDW g/dL)	PLT (x10^3^/μL)	MPV (fL)	Retic (%)		Spleen Weight (% body wt)	
**Females**																
***Mx1-Cre; Rasa3* control (12)**	6.2 ± 2.4	8.8 ± 0.6	13.2 ± 1.2	42.9 ± 1.9	48.8 ± 1.0	15.0 ± 0.9	30.7 ± 2.3		17.8 ± 0.9	2.9 ± 0.3	660 ± 75	5.8 ± 0.4		15.8 ± 1.9		0.9 ± 0.2
***Mx1-Cre; Rasa3* heterozygous (12)**	5.6 ± 1.6	9.0 ± 0.7	14.0 ± 1.0	42.7 ± 3.2	47.4 ± 0.9	15.5 ± 0.6	32.9 ± 1.6		16.7 ± 1.1	2.7 ± 0.3	620 ± 45	5.8 ± 0.4		14.6 ± 4.0		0.8 ± 0.2
***Mx1-Cre; Rasa3* mutant (17)**	3.7 ± 1.4*^#^	4.4 ± 1.5*^	7.1 ± 2.4*^	27.6 ± 8.1*^	63.6 ± 8.1*^	16.0 ± 1.0*	25.7 ± 0.3.6*^		21.2 ± 2.6*^	3.3 ± 0.6*	32 ± 27*^	12.1 ± 3.5*^		68.6 ± 17.1*^		2.5 ± 0.9*^
**Males**																
***Mx1-Cre; Rasa3* control (7)**	5.3 ± 1.4	8.2 ± 0.6	12.5 ± 0.8	39.5 2.5	48.0 ± 2.1	15.2 ± 0.5	31.7 ± 1.2		18.5 ± 1.5	2.7 ± 0.3	803 ± 2.3	5.9 ± 0.5		12.9 ± 3.9		0.7 ± 0.2
***Mx1-Cre; Rasa3* heterozygous (7)**	5.4 ± 2.3	8.8 ± 1.0	13.5 ± 1.5	43.1 ± 3.5	49.3 ± 3.2	15.4 ± 0.5	31.3 ± 1.3		17.2 ± 2.3	2.6 ± 0.4	660 ± 68^+^	5.9 ± 0.7		17.0 ± 8.8		0.6 ± 0.2
***Mx1-Cre; Rasa3* mutant (18)**	3.7 ± 1.1^#^	3.5 ± 1.2*^	5.8 ± 1.8*^	23.9 ± 7.1*^	68.2 ± 5.6*^	16.4 ± 1.5	24.1 ± 2.2*^		22.6 ± 4.6^+^^	3.7 ± 0.6^	38 ± 21*^	12.6 ± 4.6^+^^#^		57.6 ± 16.3*^		2.1 ± 0.7*^

All values X ± SD; +p < 0.05 *vs*. WT

^#^p < 0.05 *vs*. Het

*p < 0.01 *vs*. WT

^p < 0.01 *vs*. Het.

WBC, white blood cell count; RBC, red blood cell count; Hgb, hemoglobin; Hct, hematocrit; MCV, mean corpuscular volume; MCH, mean corpuscular hemoglobin; MCHC, mean corpuscular hemoglobin concentration; RDW, red cell distribution width; HDW, hemoglobin distribution width; PLT, platelet count; MPV, mean platelet volume; Retic, reticulocytes

### Bone marrow function is suppressed in *Mx1-Cre; Rasa3* mutant mice

**Fig 5:**
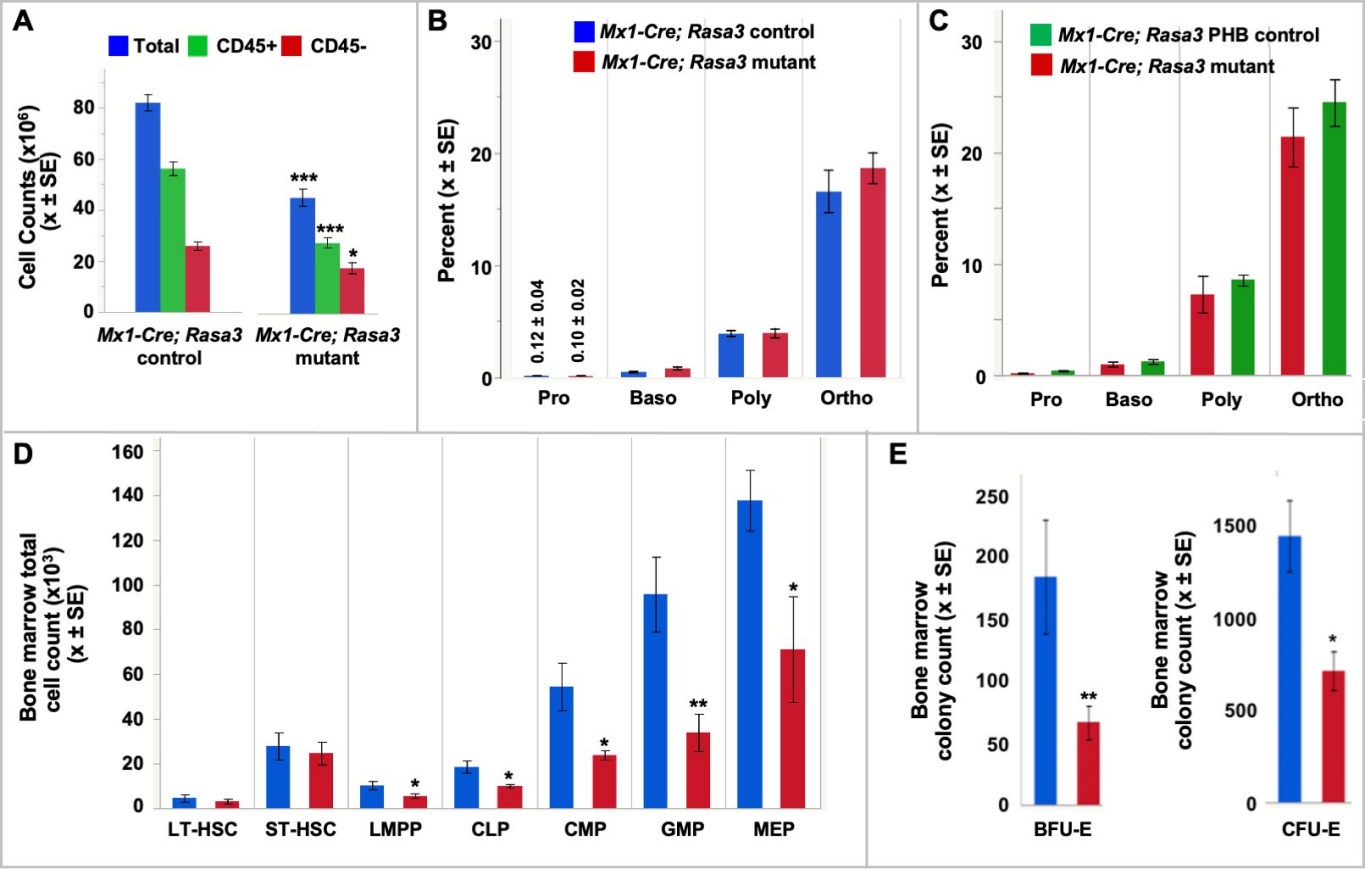
Suppression of bone marrow hematopoiesis in the absence of RASA3. **(A)** Total, CD45^-^ erythroid, and CD45^+^ non-erythroid cell counts are significantly depleted in *Mx1-Cre; Rasa3* mutant bone marrow. n = 8 **(B,C)** Terminal erythroid differentiation progresses normally in mutant bone marrow compared to both non-anemic (n = 8) and phlebotomized anemic (n = 6) controls. **(D)** Total counts of all hematopoietic progenitor cell types are significantly decreased in *Mx1-Cre; Rasa3* mutant bone marrow *vs*. control (n = 8). LT- and ST-HSCs do not significantly differ. **(E)** Decreased BFU-E and CFU-E colony formation capacity in mutant bone marrow. n = 3. Equal numbers of mutant and control were plated in colony forming assays. LT-HSC, long term hematopoietic stem cell; ST-HSC, short term hematopoietic stem cell; LMPP, lymphoid-primed multipotent progenitor; CLP, common lymphoid progenitor; CMP, common myeloid progenitor; GMP, granulocyte monocyte progenitor; MEP, Megakaryocyte-erythroid progenitor. All values X ± SEM. *p < 0.05, **p < 0.01, ***p < 0.001.

The decreased numbers of red cells, leukocytes and platelets in the *Mx1-Cre; Rasa3* mutant mice are suggestive of bone marrow failure, and reminiscent of the *scat* phenotype [13]. We observed that basal (bone marrow) erythropoiesis is suppressed in *Mx1-Cre; Rasa3* mutant mice compared to control mice; total, CD45^-^ erythroid, and CD45^+^ non-erythroid cell counts are all significantly depleted in mutant *vs*. control bone marrow (Fig 5A). Flow cytometric analyses using CD44, Ter119 and forward scatter (FSC) as markers of terminal erythroid differentiation [28] show that progression through terminal differentiation proceeds normally in *Mx1-Cre; Rasa3* bone marrow, keeping pace with both non-anemic controls and controls rendered anemic by phlebotomy (PHB), which do not differ from each other in any peripheral blood red cell parameters (Fig 5B and 5C and S3 Table). Thus, basal erythropoiesis is quantitatively suppressed but the progression from one precursor stage to the next is unaffected in the absence of RASA3.

We next determined the status of hematopoiesis at the level of HSPCs using flow cytometric approaches. The frequencies of all HSPC populations in *Mx1-Cre; Rasa3* mutant and control bone marrow are the same (S4A Fig) as is the absolute number of LT- and ST-HSCs (Fig 5D). However, the absolute number of all progenitors is significantly decreased in mutant bone marrow compared to control (Fig 5D). Moreover, in keeping with reduced progenitors including megakaryocyte-erythroid progenitors (MEPs), the functional capacity of mutant bone marrow to produce BFU-E and CFU-E colonies *in vitro* is strikingly reduced (Fig 5E), all of which would be predicted to contribute to pancytopenia in the absence of RASA3. These data suggest that RASA3 impacts the number of progenitors in the bone marrow during hematopoiesis.

### Spleen stress erythropoiesis fails to compensate for RASA3 deficiency

**Fig 6:**
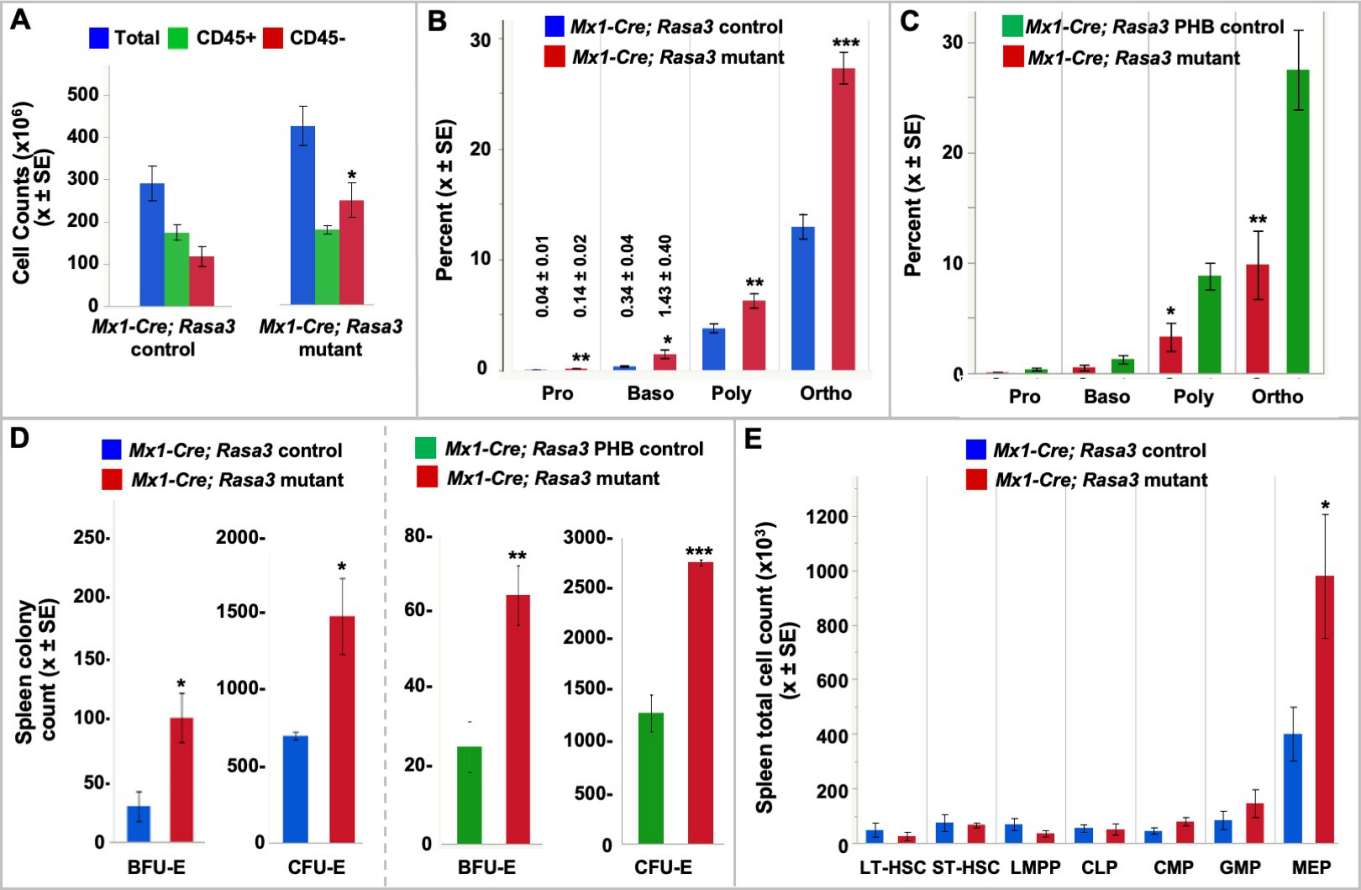
Blunted spleen stress erythropoiesis in the absence of RASA3. **(A)** Total CD45^-^ erythroid cells are significantly increased in *Mx1-Cre; Rasa3* mutant adult spleen. n = 8. **(B)** Terminal differentiation in the mutant spleen is accelerated compared to *Mx1-Cre* negative control mice, showing increased precursors at all stages. n = 8. **(C)** Stress erythropoiesis in *Mx1-Cre; Rasa3* mutant spleen is dramatically blunted compared to *Mx1-Cre* control mice rendered anemic by phlebotomy (PHB). n = 6. **(D)** Increased BFU-E and CFU-E colony formation in mutant spleen. n = 3. Equal numbers of mutant and control were plated in colony forming assays. **(E)** Megakaryocyte-erythroid progenitors are increased in number in *Mx1-Cre; Rasa3* mutant spleen; no other significant differences are seen. n = 8. LT-HSC, long term hematopoietic stem cell; ST-HSC, short term hematopoietic stem cell; LMPP, lymphoid-primed multipotent progenitor; CLP, common lymphoid progenitor; CMP, common myeloid progenitor; GMP, granulocyte monocyte progenitor; MEP, Megakaryocyte-erythroid progenitor. All values X ± SEM. *p < 0.05, **p < 0.01, ***p < 0.001.

Under normal conditions, the spleen is not a significant source of erythroid cells in adult mice. However, under anemic stress, spleen erythropoiesis increases significantly resulting in as much as a 100-fold increase in the number of erythroblasts that markedly increases overall spleen size [28–30]. Accelerated erythropoiesis under anemic stress also leads to increased peripheral blood reticulocytes. In *Mx1-Cre; Rasa3* mutant mice, stress erythropoiesis is established within two weeks of induction of anemia; the spleen is grossly enlarged (Table 1) and its normal nodular architecture is effaced by expansion of the red pulp (S5 Fig). The total cell number is increased in mutant spleen compared to control (p = 0.0531) and this increase reflects expansion of the erythroid compartment, as CD45^-^ erythroid but not CD45^+^ non-erythroid cells are significantly increased compared with controls (Fig 6A).

Flow cytometric analyses of terminal erythroid differentiation reveal an increase in all precursor stages from pro- to orthochromatic erythroblasts in *Mx1-Cre; Rasa3* mutant compared to control spleen (Fig 6B). The spleen erythropoietic response in *Mx1-Cre; Rasa3* PHB anemic controls, however, was substantially higher than in *Mx1-Cre; Rasa3* mutants (Fig 6C). Thus, while comparison to control mice suggests that a stress response is initiated in *Mx1-Cre; Rasa3* mutant spleen, comparison to PHB control mice reveals that the response is strikingly less robust than that seen in anemic mice in which the *Rasa3* locus is intact. Moreover, since the reticulocyte response from phlebotomy originates from the precursor pool [31–33], these data suggest that the stress erythropoietic response in *Mx1-Cre; Rasa3* mutants is initiated earlier, at the progenitor level. Colony-forming assays on *Rasa3* mutant, anemic PHB controls, and non-anemic controls support this hypothesis. BFU-E and CFU-E colony numbers are significantly increased in *Rasa3* mutant spleen compared to both non-anemic and anemic (PHB) controls, which are roughly comparable to each other (Fig 6D). Moreover, MEPs are increased both in number and in frequency in *Mx1-Cre; Rasa3* mutant spleen compared to control, although no other significant differences are seen in HSPC populations (Fig 6E and S4B Fig). Together, our results support the hypothesis that although deletion of *Rasa3* leads to an increase of early erythroid progenitors in the spleen, both in number and function, enhanced erythropoiesis is not propagated effectively through terminal differentiation.

### Genetic background profoundly influences the *Rasa3* mutant phenotype

**Fig 7:**
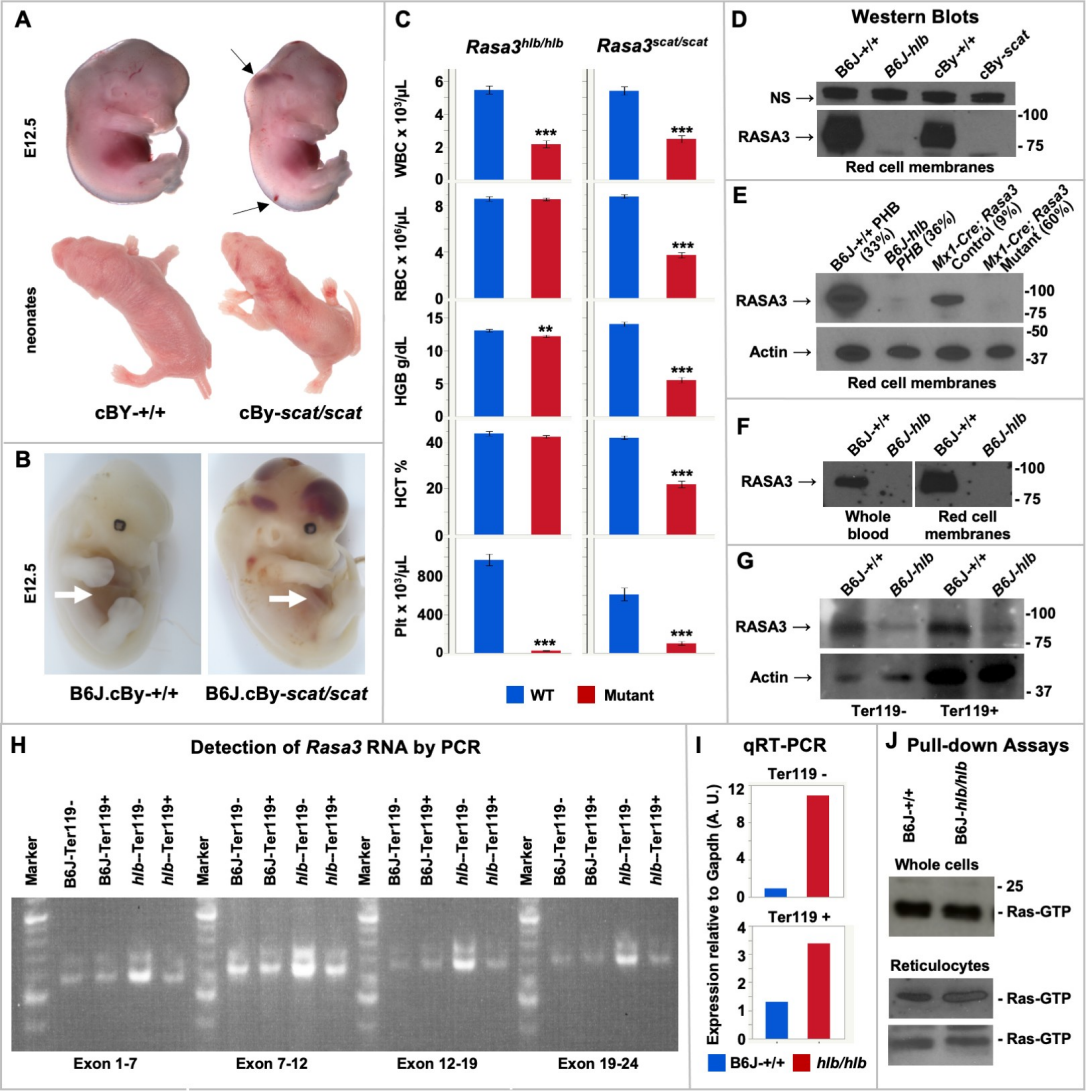
Effects of genetic background and molecular variant on RASA3 mutant phenotype. **(A)** E12.5 (top) and neonatal (bottom) BALB/cBy (cBy) control and *scat/scat* mice. Pallor and extensive bruising are consistently observed in newborn *scat* homozygotes. **(B)** Congenic B6J.cBy *scat* homozygote displaying extensive hemorrhaging and a strikingly pale fetal liver (arrows) *vs*. control. Examination of multiple litters from E12.5 to E14.5 showed no live B6J-*scat/scat* mice. **(C)** *Hlb381* homozygotes (*hlb/hlb*) carrying missense mutation H794L are leukopenic and thrombocytopenic but differ markedly from *scat/scat* mice (G125V) in that severe anemia is absent. S2 and S5 Tables provide complete blood count data for *scat* and *hlb381*, respectively, at 3–4 weeks of age. All values X ± SEM. **p <0.01, ***p < 0.001 *vs*. strain specific WT control. **(D-G)** RASA3 Western blots: **(D)** control and homozygous mutant *hlb* and *scat* red cell membranes (NS = non-specific band serving as loading control from same blot), **(E)** phlebotomized (PHB) control and mutant *hlb* and *Mx1-Cre* red cell membranes (number in parentheses = circulating reticulocyte percentage), **(F)** control and *hlb* whole blood and red cell membranes, and **(G)** purified control and *hlb* Ter119^-^ and Ter119^+^ spleen cells. **(H)** PCR detecting cDNA across all exons in control and *hlb* Ter119^-^ and Ter119^+^ spleen cell mRNA. **(I)** qRT-PCR reveals increased *Rasa3* expression in *hlb* Ter119^-^ and Ter119^+^ spleen cells compared to control. **(J)**. Pull down assays reveal normal levels of active Ras-GTP in *hlb* whole blood and purified reticulocytes (2 representative blots of reticulocytes shown).

Dramatic phenotypic differences are observed in engineered germline *Rasa3* null mice on the B6J genetic background compared to *scat* mice carrying a missense mutation on BALB/cBy (cBy). While a subset of cBy-*scat* mice dies *in utero*, 10–15% are born and show a characteristic pale, bruised phenotype as neonates (Fig 7A). We generated a germline B6,129 *Rasa3* null allele by crossing *Rasa3*^*fl*/+^ mice to *CMV-Cre* transgenic mice (S1 Fig). No *Rasa3*^-/-^ pups were obtained at birth from intercrosses of *Rasa3*^+/-^ mice, which appeared normal in all respects, while 16 *Rasa3*^+/-^ (57%) and 12 *Rasa3*^+/+^ (43%) progeny were obtained. Thus, on either a mixed B6,129 or a pure inbred B6NJ (S3 Fig) genetic background, *Rasa3* null mice are 100% embryonic lethal. To further investigate the effects of genetic background, we examined complete blood counts in B6NJ adults. B6NJ *Mx1-Cre; Rasa3* null mice display severe anemia, thrombocytopenia and leukopenia, closely mirroring the B6;129 *Mx1-Cre; Rasa3* null phenotype (S4 Table). We next transferred the *scat* missense allele to the B6J background to create a fully congenic line, B6J.cBy-*scat*. In this case, a dramatically different phenotype emerged in B6J congenic *vs*. cBy *scat* mic*e* despite both carrying the same missense allele. No affected offspring showing the easily recognizable *scat* phenotype at birth were identified in B6J.cBy-*scat*/+ intercrosses at any outcross generation. Genotyping of 154 neonates at the most recent outcross generation (N = 21) revealed 106 (68.8%) *scat*/+ and 48 (31.2%) wildtype mice; no *scat/scat* mice were detected, confirming 100% *in utero* lethality. Examination and genotyping of 64 congenic fetuses at E12.5-E14.5 revealed the expected 25% *scat* homozygotes; all exhibited evidence of severe bleeding with strikingly pale fetal livers (Fig 7B). Thus, congenic B6J.cBy-*scat* homozygotes differ markedly from cBy-*scat/scat* and mimic germline B6J/B6NJ null mutations, confirming a striking effect of genetic background.

### Dramatic effects of molecular variant on disease phenotype

Previously, we identified the recessive N-ethyl-N-nitrosourea (ENU) induced missense mutation in *Rasa3* (H794L) in a forward genetic mutagenesis screen in B6J mice [15,34]. The mutation lies in the C-terminal 80 residue region of RASA3 that does not show homology to any known protein domains. Phenotypically the mutant model, designated *hlb381*, differs dramatically from *Rasa3* germline null, cBy-*scat* and congenic B6J.cBy-*scat* homozygotes. *Hlb381* homozygotes are born at the expected Mendelian ratio and survive normally. No pallor or evidence of bruising has ever been noted at birth or postnatally. Complete blood counts reveal severe thrombocytopenia and leukopenia at birth and throughout life. Unlike cBy-*scat* (G125V), however, severe anemia is not present, although some evidence of a mild compensated anemia (increased spleen weight and circulating reticulocyte percentage) is seen at 3–4 weeks of age (Fig 7C and S5 Table). This is most evident in males and is largely resolved by 6 weeks of age (S6 Table). The spleen architecture is normal, with clearly delineated white and red pulp areas (S6 Fig). Thus, different *Rasa3* missense alleles, G125V (B6J.cBy-*scat*) and H794L (B6J-*hlb381*), confer dramatically different phenotypes on the same (B6J) genetic background, reinforcing the significant structure-function differences between these RASA3 amino acid variants.

Membrane localization of RASA3 is required for GAP activity to downregulate RAS and RAP activity [1,4]. RAS, but not RAP, is present in mouse erythrocytes; RAP is absent in mouse red cells but abundant in platelets [13,15,35]. Loss of RASA3 activity would be predicted to increase active GTP-bound RAS and RAP. Indeed, previous studies in *hlb381* revealed increased RAP-GTP in platelets, leading to spontaneous platelet activation and markedly increased clearance of circulating platelets [15]. We also showed previously that active RAS-GTP is increased in RASA3 mutant *scat* reticulocytes as a result of mislocalization of RASA3 to the cytosol leading to abnormal erythropoiesis [13]. The lack of significant anemia in *hlb381* led us to hypothesize that, unlike G125V in *scat*, H794L does not affect membrane localization and/or RAS activation. To test this hypothesis, we performed western blots and pull-down assays. Surprisingly, although easily detectable in B6J controls, RASA3 is undetectable in *hlb381* red cell membranes even upon over-exposure of ECL western films (Fig 7D). RASA3 is most abundant in reticulocytes, but it declines rapidly via the exosomal pathway during reticulocyte maturation [13]. However, even when significant reticulocytosis is induced via phlebotomy, RASA3 remains undetectable in *hlb381* red cell membranes (Fig 7E) and whole cells (Fig 7F). Only when we examined Ter119^-^ erythroid progenitors and Ter119^+^ erythroid precursors cells isolated from the spleen by flow-cytometry were we able to detect RASA3 protein in *hlb381*, albeit at lower levels than controls (Fig 7G). Notably, prior studies in platelets showed that RASA3 protein is also reduced in homozygous *hlb381* platelet lysates to levels comparable to those in platelets from heterozygous mice carrying a RASA3 null allele, suggesting that the H794L allele markedly impairs RNA expression or RASA3 protein stability [15]. Our findings here confirm instability of the H794L RASA3 protein, as conventional PCR analysis of spleen erythroid cells detects cDNA for all *Rasa3* exons (Fig 7H). Moreover, significantly more PCR product is seen for *hlb381* phlebotomized spleen erythroid cells, particularly Ter119^-^ erythroid progenitors, compared to WT; qRT-PCR confirms increased *Rasa3* expression in *hlb381* homozygotes (Fig 7I).

Finally, in contrast to previous studies showing increased RAP-GTP in *hlb381* platelets [15], pull-down assays failed to show consistently increased active RAS-GTP in *hlb381* erythroid cells, notably in purified reticulocyte fractions (Fig 7J). This is a surprising result given the absence of RASA3 in *hlb381* reticulocytes and mature red cells and the easily detectable increase in RAS-GTP seen in *scat* cells [13]. There are two possible explanations for the dramatic and varied mutation effects of G125V and H794L: (a) the reduced amounts of RASA3 in erythroid progenitors and precursors suffice to maintain normal RAS-GTP levels in *hlb381* reticulocytes and mature red cells or, (b) genetic modifier genes present in the B6J genetic background but absent in cByJ compensate for loss of RASA3 in *hlb381*. To further elucidate the mechanisms leading to these dramatic mutation effects will require further study. Significantly, it is clear that while G125V affects both RAS and RAP activity [13,15,17], leading to pancytopenia, H794L primarily affects RAP activity, as shown here and in previous work [15], largely sparing red cells and providing an explanation for absence of significant anemia in *hlb38*1. Additional mechanisms cannot be excluded at this time.

### Analyses of global transcriptomes in *scat* suggest a primary role of spleen in disease genesis and differentiate potential mechanisms underlying crisis *vs*. partial remission

**Fig 8:**
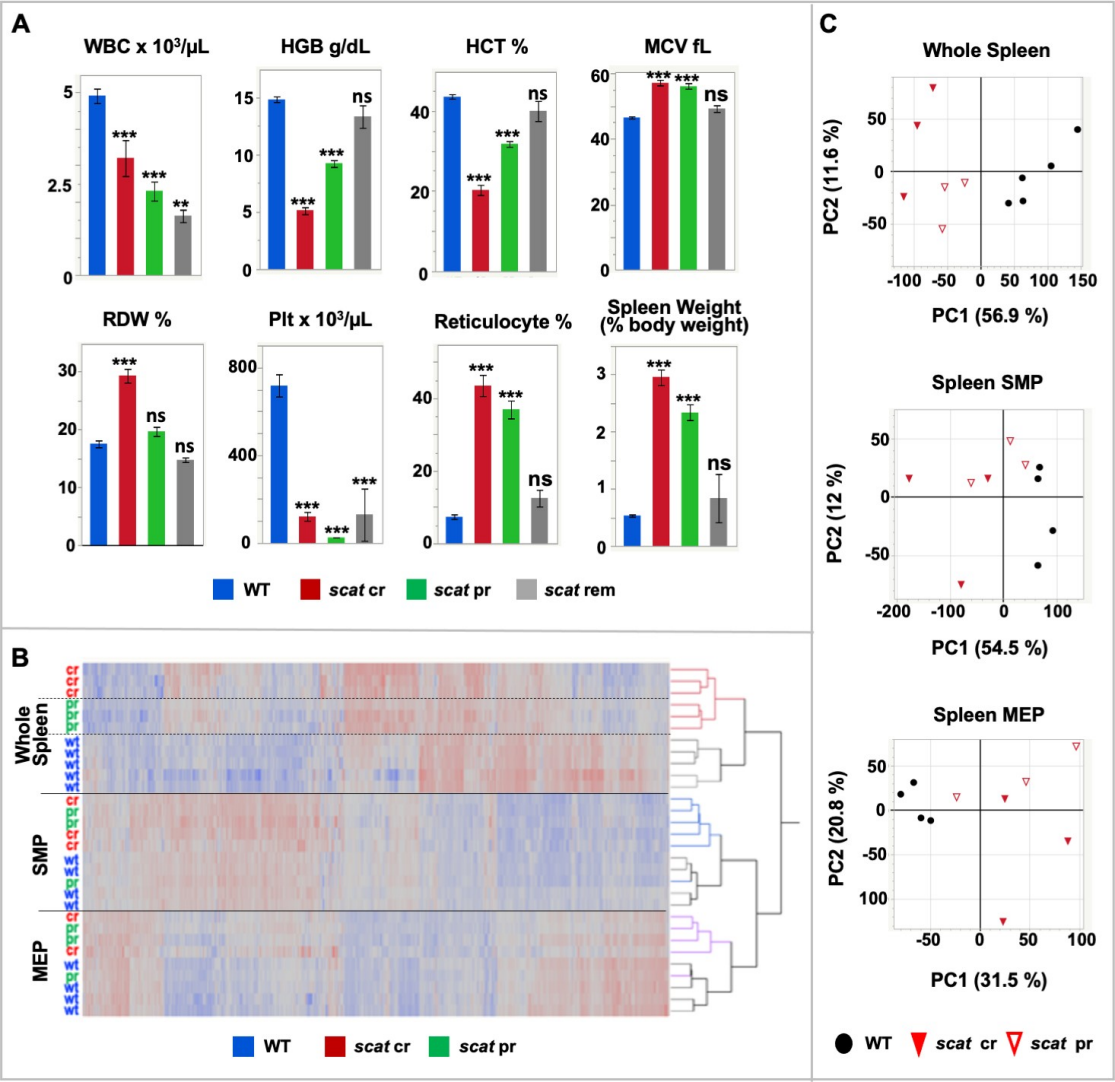
Transcriptome analyses in *scat* mice. **(A)** Blood counts in control (WT, n = 38), *scat* crisis (cr, n = 25), *scat* partial remission (pr, n = 22) and *scat* full remission (rem, n = 4) and spleen weights (n = 24, 19, 12, and 2, respectively). Data are male and female combined. All values X ± SEM. **p < 0.01, ***p < 0.001, ns not significant. **(B)** Hierarchal clustering of expression differences clearly separates whole spleen, SMP, and MEP samples (solid lines) and WT, cr and pr within whole spleen (dashed lines). **(C)** Principle component analysis.

To determine global transcriptome changes in RASA3 defective hematopoietic tissues and cells, we performed RNAseq using 3–5 biological replicates of whole spleen and bone marrow and flow cytometry sorted HSPC populations from *scat* homozygotes and their WT littermates (S7 Table). Furthermore, the cyclic nature of *scat* disease progression, in which rare full remissions and, more commonly, partial remissions occur (Fig 8A), allowed us to parse the mutant samples into cr and pr and compare differential expression in each to WT. Mice were classified as pr based on erythroid parameters intermediate to WT and cr with the exception of the reticulocyte percentage and spleen weight, which are expected to remain high during recovery from anemia. Complete blood counts for mice in each group used for RNAseq are given in S8 Table. Flow cytometry sorted populations obtained were megakaryocyte-erythroid progenitors (MEP) and so-called “stem and myeloid progenitors” (SMP). Because Sca1 (*Ly6a*) is expressed at very low levels in BALB mice [36], HSCs cannot be separated reliably from common myeloid progenitors. Therefore, the Lin^-^Sca^-^Kit^+^CD34^+^ CD16/32^lo^ SMP population was used for these studies [37]. Differentially expressed gene (DEG) lists are given in S1 Data.

Hierarchal clustering of DEGs in spleen and spleen-derived erythroid HSPCs clearly separates whole organ, SMP, and MEP populations (Fig 8B). Moreover, in whole spleen there is a clear delineation of all three phenotypes (*scat* cr, *scat* pr, WT). Separation of the three phenotypes is not maintained in SMP and MEP cells, although the two major genotypes (*scat* and WT) are largely separated in the SMP and MEP populations with the caveat that one pr sample clusters with WT (Fig 8B). Principle component analysis (PCA) reveals that the first principle component (PC1) dominates and segregates *scat* from WT in all spleen-derived samples (Fig 8C). All WT samples cluster together—to the right of all *scat* samples in whole spleen and SMP and to the left in MEP samples. Thus, genotype accounts for 57%, 55%, and 32% of the expression variation between *scat* and WT in whole spleen, spleen SMP and spleen MEP samples, respectively. In whole spleen PC1 additionally segregates cr from pr suggesting that functionally distinct mechanisms are at play among the three phenotypes in the spleen (Fig 8C). In bone marrow, PC1 separates *scat* from WT in whole bone marrow only and does not distinguish cr and pr (S7 Fig). Overall, these analyses indicate a primary role for expression variation in spleen-derived populations in driving the phenotypes observed.

To explore functions associated with altered expression patterns we performed gene ontology (GO) analysis using DAVID [38]. Many comparisons of expression differences are possible between the many datasets generated; here, we present those we found to be most informative. Additional comparisons can made using DEG lists provided (S1 Data). Examination of differential expression in whole spleen and bone marrow provides a snapshot of the physiological effect of mutant RASA3 in the context of the whole animal with the caveat that the disease process includes alteration of organ cellular composition in *scat* (as shown in Figs 5A and 6A and [13]). When spleen DEGs for all mutants (cr and pr combined) are compared to WT controls using GO terms for Biological Process, terms associated with cell cycle and immune function predominate; cell cycle terms dominate genes upregulated in mutants, and immune function down-regulated (S8 Fig). Notably, immune cell changes [13] have been documented in *scat*, and cell cycle changes are seen in terminally differentiating erythroid precursors at the basophilic and polychromatophilic stages [27]. Similar comparisons of bone marrow DEGs are dominated by immune and chromatin-associated terms (S8 Fig).

Examination of DEGs in flow-sorted SMP and MEP populations provides a view of the effects of altered expression at the cellular (stem and progenitor) level. In spleen SMPs (S9 Fig), immune-related GO terms completely dominate when datasets for all DEGs and for downregulated genes are compared, similar to whole spleen. The same is seen in spleen MEPs (S10 Fig). Cell cycle terms did not appear in the downregulated genes, however, for either SMPs or MEPs, suggesting that cell cycle changes are restricted to more mature cells of the spleen such as erythroid precursors. When GO terms redundant for immune-related processes are filtered out of SMP datasets, additional terms related to cytokines, adhesion, signaling and signal transduction, and apoptosis are significant as well (S9 Fig). GO terms for genes upregulated in mutant spleen SMPs include heme synthesis, ion transport, and apoptosis. In spleen MEPs, terms related to phosphorylation are significant in genes whose expression is downregulated in mutants (S10 Fig).

Differential expression in bone marrow SMPs reveals terms related to transport, signaling and proliferation (S9 Fig). Notably, genes upregulated in mutant bone marrow SMPs are enriched for GO terms related to ion homeostasis and transport. As only 27 genes were downregulated in mutant bone marrow SMPs, no GO terms reached significance for this group. In bone marrow MEPs (S10 Fig) GO terms for signaling, signal transduction and cytokines predominated. In MEPs, no significant GO terms emerged from the upregulated dataset.

**Fig 9:**
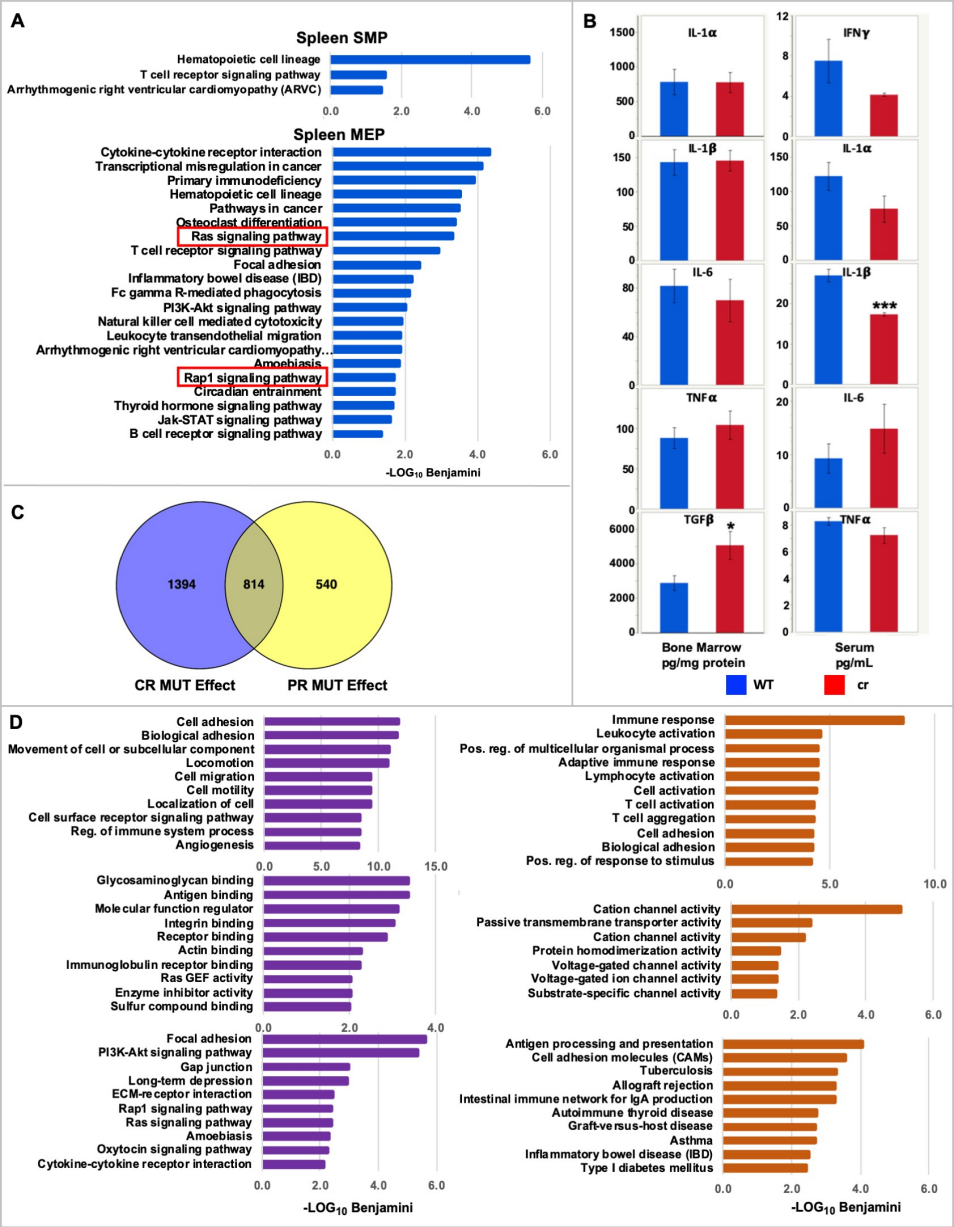
Gene ontology analyses of differentially expressed genes. **(A)** Significant pathways (KEGG) associated with spleen SMP and MEP differential expression. **(B)** Cytokine levels in bone marrow fluid and serum. **(C)** Venn diagram of expression differences across all cells to show mutation (MUT) effect when all cells and tissues are combined. Blue, DEGs in *scat* cr *vs*. WT. Yellow, DEGs *scat* pr *vs*. WT. **(D)** Significant biological process (top), molecular function (middle) and KEGG pathways (bottom) exclusive to cr (left, purple) and pr (right, orange) DEGs.

Analysis of KEGG pathways in DAVID, which point to pathways in which DE genes are statistically over-represented and thus can provide clues to underlying mechanisms, were informative in spleen SMP and MEP (Fig 9A). In SMP DEGs, the arrhythmogenic right ventricular cardiomyopathy (ARVC) pathway is significant. A closer look at the DEGs associated with this pathway reveals differential expression of genes encoding components of adherens junctions (AJ) and desmosomes. Notably, the original description of RASA3 knockout noted underdeveloped AJs between capillary endothelial cells [12]. Identification of these differentially expressed genes in SMPs was unexpected. Endothelial cells arise from hemangioblasts, which are thought to be restricted to the embryo. Nevertheless, the data indicate aberrant expression of genes critical to vascular integrity and are consistent with the RASA3 deficient phenotype. In similar analyses of spleen MEP DEGs, RAS and RAP signaling were among significant pathways (Fig 9A). Cytokine interactions also emerged. A preliminary survey of a small subset of cytokines in bone marrow fluid showed increased TGFβ and, in serum, decreased IL-1β (Fig 9B).

Given that GO terms and KEGG pathways known to be altered in the *scat* disease phenotype from previous studies [13,15–17] emerged in the above analyses of differential gene expression (e.g., signal transduction, RAS and RAP signaling pathways, immune processes), as well as some unexpected terms (ion homeostasis/transport), we asked if novel processes associated with recovery of anemia could be identified by examination of *scat* pr and cr datasets compared to WT individually. We analyzed differential expression in cr vs. WT and pr vs. WT across all samples combined to determine an overall mutation effect independent of tissue/cell origin, which increased statistical power significantly. Venn diagrams reveal 540 DEGs unique to pr, and 1384 unique to cr (Fig 9C)[39]. Significant GO terms for Biological Process, Molecular Function, and KEGG pathways unique to cr and pr DEGs are shown in Fig 9D. Importantly, examination of these terms reveal that those exclusive to cr conform to our expectations to a large degree given what is known about the functions of RASA3 and the *scat* crisis phenotype from this and prior studies [13,15], providing confidence in our approach. Terms and processes exclusive to pr provide hypothesis-driving new directions for future studies. Notably, the most significant and novel Molecular Function GO terms that emerged are all related to channel activity, suggesting that changes in red cell transporter activity may contribute to partial recovery of the *scat* anemia.

### *RASA3* knockdown leads to impaired human terminal erythroid differentiation

**Fig 10:**
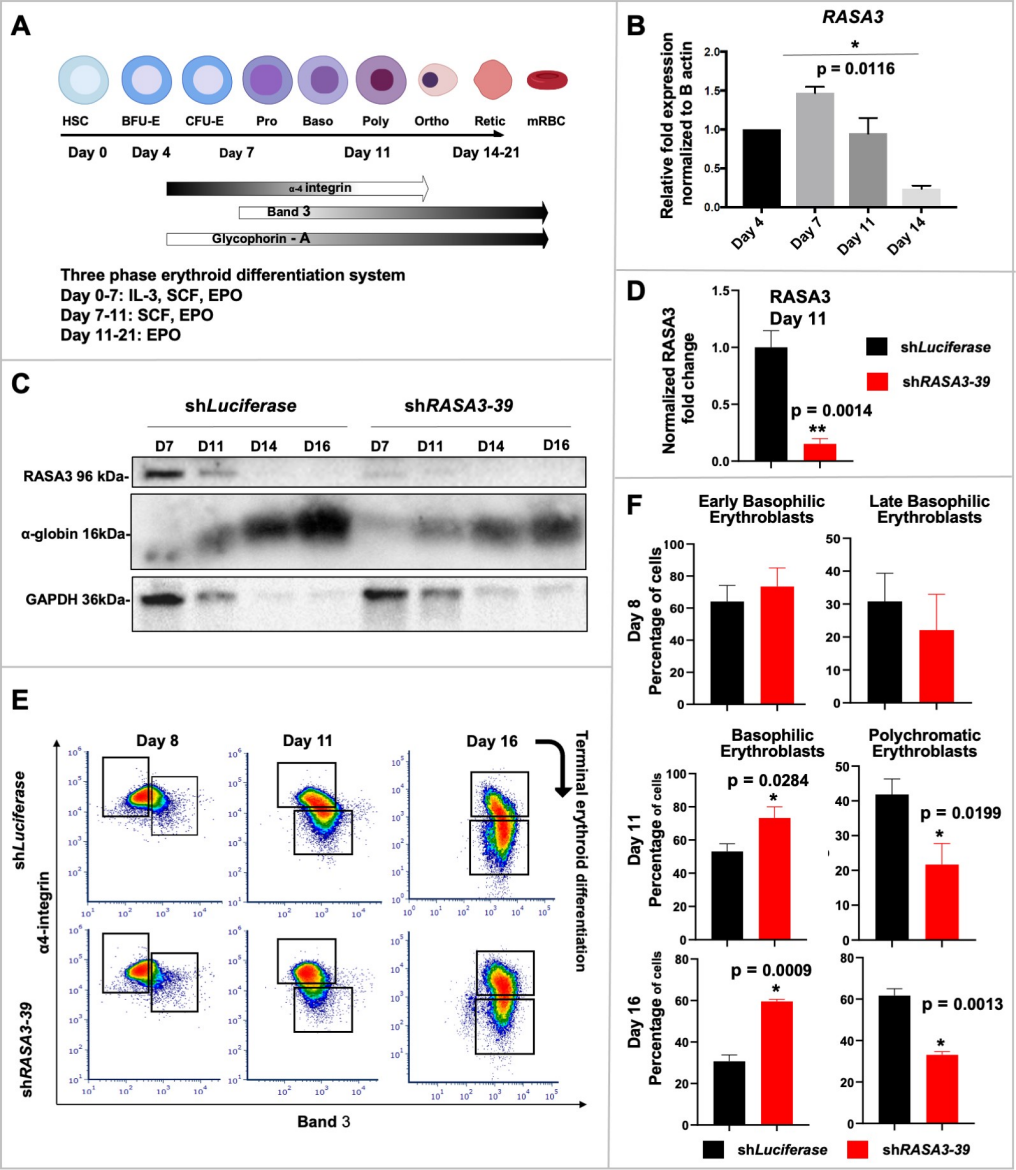
RASA3 plays a role in human terminal erythroid differentiation in vitro. **(A)** Schematic of the three-phase erythroid differentiation culture system utilized with cord blood-derived CD34+ cells. **(B)** qRT-PCR demonstrates *RASA3* kinetics of expression during human erythropoiesis. **(C)** RASA3 expression levels in sh*Luciferase* control versus knockdown cultures. **(D)** Quantification of shRASA3-39 mediated knockdown of RASA3 (n = 4). **(E)** Representative flow cytograms of terminal erythroid differentiation of glycophorin A (GPA) positive cells, monitored by progressive loss of α4-integrin and acquisition of Band 3, in shRASA3 versus shLuciferase through Day 16. Boxes represent early (top) and late (bottom) cells quantified in F. **(F)** Quantification of erythroblast population percentages in sh*Luciferase* and shRASA3-39 at days 8, 11, and 16 in vitro (n = 3, 7, 3, respectively).

Despite the dramatic phenotype leading to bone marrow failure in *scat*, so far there has been no report of human patients with a mutation in *RASA3* leading to similar conditions. After characterizing the role of RASA3 in murine erythropoiesis, we next investigated the potential role of RASA3 in human erythropoiesis to confirm the relevance of the RASA3-Ras axis to inherited bone marrow failure syndromes. To do so, we used cord blood-derived CD34+ cells and differentiated them toward reticulocytes using a 3-phase erythroid differentiation culture system (Fig 10A)[40]. Using qRT-PCR and western blotting we observed that RASA3 was expressed during human erythropoiesis (Fig 10B and 10C, left panel). To assess the role of RASA3 during human erythropoiesis, we used lentiviral knockdown to silence RASA3. Two shRNA constructs were each compared to sh*Luciferase* controls (Figs 10D and S11). We verified the knockdown efficiency by western blot (Fig 10C, right panel) and RASA3 expression was significantly decreased by more than 80% by Day 11 of culture (Fig 10D). We then monitored terminal erythropoiesis using Glycophorin A, alpha4-integrin and Band3 as surface markers [41] and noticed a delay in sh*RASA3* cultures from days 11–16 in culture, represented by delayed loss of α4-integrin and delayed acquisition of Band-3 (Fig 10E, bottom panel). sh*RASA3* cultures consistently had significantly higher proportions of earlier populations as identified by these markers compared to sh*Luciferase* cultures, quantified in Fig 10F. This delay corresponds to the late basophilic, polychromatic, and orthochromatic erythroblast stages and aligns with the erythroid phenotype observed in *scat*, reinforcing the importance of RASA3 in late erythroid differentiation. Loss of RASA3 did not alter erythroblast proliferation *in vitro* (S12 Fig), highlighting a unique role for RASA3 in terminal erythroid differentiation.

## Discussion

Studies in the *scat* mutant mouse model, characterized by severe anemia, thrombocytopenia and leukopenia, and confirmed in zebrafish by morpholino knockdown, firmly established a critical non-redundant role of RASA3 in vertebrate blood formation [13]. The original gene knockout study in which a catalytically inactive form of RASA3 was produced [12] showed that germline loss of RASA3 activity results in extensive hemorrhage, a phenotype that was attributed to quantitatively decreased adherens junctions between endothelial cells. Subsequent studies confirmed that the RASA3-RAP axis is critical in endothelial cell adhesion and vessel development [16]. *Scat* mutants carrying the G125V missense mutation also show evidence of hemorrhage, although less severe, *in utero* (Fig 7A), which may account for the fact that *scat*/+ intercrosses produce 10–15% homozygous *scat* newborns *vs*. the expected 25%. Postnatally, cranial hemorrhage frequently develops during crisis episodes in *scat* [13,14]. Interestingly, vascular malformations in the brain accompanied by hemorrhagic stroke were described recently in mice expressing activated HRAS alleles [42]. Whether such a mechanism underlies cranial bleeds in *scat* awaits further study.

Here we show that *Rasa3* null embryos not only show massive bleeding but evidence of insufficient fetal liver (small, pale) erythropoiesis as well. The fetal liver functions as the major source of hematopoiesis during development; fetal liver erythropoiesis expands exponentially between E12.5 and E16.5 and imparts the red color visible at this time [43]. Significantly, this phenotype is also observed in conditional *Vav-Cre*; *Rasa3* mutant embryos, indicating a requirement for RASA3 in the embryo to maintain optimal erythropoiesis and maintenance of vascular integrity. To investigate hematopoiesis further, we first focused on terminal erythroid differentiation, as a pronounced block at the poly- and orthochromatophilic stages was previously documented in *scat* [13]. Surprisingly, no phenotype emerged in *Epor-Cre; Rasa3* mutants, and RASA3 protein persisted in erythroid cells suggesting that RASA3 produced in progenitors maintained erythroid terminal differentiation. Indeed, when *Rasa3* is deleted in adults using *Mx1-Cre*, the severe, pancytopenic *scat* phenotype emerges. Bone marrow hematopoiesis is clearly suppressed; total cellularity is decreased, all progenitors are significantly decreased in number, and the functional capacity to produce erythroid progenitors (BFU-E, CFU-E) is significantly decreased (Fig 5).

In addition to suppressed bone marrow function in *Mx1-Cre*; *Rasa3* mutants, stress erythropoiesis in the spleen is ineffective, as has been described previously in *scat* mice [13]. Although increased numbers of all red cell precursors are produced in the *Mx1-Cre*; *Rasa3* mutant spleen compared to non-anemic controls, their numbers fall far short of those produced in PHB anemic control mice in which RASA3 is intact. Notably, unlike the *scat* model, terminal erythropoiesis is not delayed at any stage in *Mx1-Cre*; *Rasa3* mutants. However, the models differ in many respects including their ages and the nature of the RASA3 defect as well as the disease itself, i.e., chronic vs. acute. Although terminal erythroid differentiation in *Mx1-Cre*; *Rasa3* mutant spleen falls short of PHB anemic controls, BFU-E and CFU-E colony forming ability is significantly greater (Fig 6) than both anemic and non-anemic controls. Thus, the *Mx1-Cre*; *Rasa3* mutant spleen initiates a stress response at the progenitor level that fails to be propagated through the precursor stages where rapid amplification of red cell production would occur.

The phenotypic differences amongst various RASA3 mouse models are striking [13,15–17,44]. To address this aspect of RASA3 function, we backcrossed the *scat* mutation onto the B6J genetic background, producing a fully congenic strain. Remarkably, 100% of B6J-*scat/scat* congenic mice die *in utero* (E12.5–13.5) recapitulating the germline null phenotype. Thus, genetic modifiers differing between strains cByJ and B6J dramatically alter the effects of the RASA3 G125V mutation. Extensive quantitative trait mapping will be required to identify these modifier genes. The molecular variant also has a profound impact, as exemplified by the chemically induced *hlb381* model carrying a missense mutation, H794L, near the C-terminus of RASA3 in a region not structurally related to any known protein domains. *Hlb381* is on the B6J genetic background but survives normally, unlike B6J congenic *scat* mice. Previously, we showed that RASA3 protein levels were decreased in *hlb381* platelets, suggesting that the mutant protein was unstable. We also showed that as a consequence of decreased RASA3, active RAP-GTP levels increased in *hlb381* platelets [15]. Here, despite significantly increased mRNA expression, RASA3 protein is not detected in unfractionated circulating *hlb381* whole cells, red cell membrane ghosts or purified reticulocytes, but is detected at much reduced levels in purified spleen Ter119^+^ and Ter119^-^ cells, confirming instability of RASA3 H794L (Fig 7).

Decreased stability of RASA3 H794L leading to its absence on the membrane as well as in the cytosol in *hlb381* reticulocytes and mature red cells would predict that active RAS-GTP with its consequent aberrant influence on erythropoiesis would arise in *hlb381* mice, as has been described in mutations in which RAS is constitutively activated, including *scat* [9,13,45]. However, we failed to show consistently increased active RAS-GTP in *hlb381* erythroid cells, indicating that absence of RASA3 does not significantly alter RAS activation and perhaps accounting, at least in part, for the absence of significant anemia in *hlb381*. The data in total suggest that mechanisms in addition to failure of mutant RASA3 to bind the membrane in *scat* cells and/or currently unknown compensatory mechanisms due to genetic modifiers operating in *hlb381* influence hematopoiesis in mouse models of *Rasa3* defects. The data further suggest that G125V alters RASA3’s GAP activity for both RAS and RAP, while H794L primarily affects RAP activation. The influence of genetic background and molecular variant is reflective of phenomena in inherited bone marrow failure syndromes (IBMFS). In human IBMFS patients, the same mutation can lead to disparate phenotypes even among relatives, suggesting effects of both genetic background and epigenetic modification, and distinct variants in a given IBMFS gene can be associated with varied disease phenotypes [46,47].

Of the RASA3 deficient models described to date, only *scat* shows a variable phenotypic progression of crisis (cr) and partial remission (pr). We exploited this aspect of the *scat* disease progression to examine global transcriptome differences between WT, cr, and pr HSPCs (SMP, MEP) and whole tissues (spleen and bone marrow). Analyses of differential expression suggests a primary influence of the spleen on disease progression. Hierarchal clustering separated expression differences by cell/tissue type derived from both spleen and bone marrow, but only in whole spleen did the three phenotypes (WT, cr, pr) partition into distinct clusters (Fig 8). Principle component analysis confirmed a striking phenotype effect on differential expression in whole spleen and in spleen SMPs and MEPs (Fig 8). In all three sample populations PC1 separates by genotype—*scat* and WT cluster separately. Moreover, in whole spleen, PC1 distinguishes phenotype (cr vs. pr) (Fig 8). Analysis of functional annotation GO terms unique to pr reveals unexpected differences compared to cr in that the most significant terms all related to cation and voltage channel activity (Fig 9), suggesting a novel direction for further study. Of note, multiple ion transport pathways are active in red membranes and are associated with anemia [48]. For example, defects in the calcium activated Gardos channel (KCNN4) and the mechanosensitive cation channel (PIEZO1) are known to cause hereditary xerocytosis, an autosomal dominant hemolytic anemia [49,50]. Moreover, altered activities of the Gardos channel activity and several K-Cl cotransporters exacerbate sickle cell disease and **β**-thalassemia [51,52]. Future studies aimed at determining activities of these and other transporters in *scat* red cells will shed light on their possible contribution to the *scat* disease.

shRNA-mediated knockdown of RASA3 in cord blood-derived CD34+ cells revealed that the role of RASA3 in terminal erythroid differentiation is at least partially conserved in humans. The conservation of the differentiation delay across murine models and the human system reinforces the importance of RASA3 in processes of mammalian terminal erythroid differentiation. Elucidation of the role of RASA3 in human erythroid progenitors was limited by the technical timeline of lentiviral knockdown of primary CD34+ cells. These cells are transduced on day 2 of culture and selected by puromycin beginning on day 4, allowing first analyses at day 7, which corresponds to the proerythroblast stage.

A key difference between the murine models and the human *in vitro* model is the type and consequence of *RASA3* mutation. In *scat*, RASA3 is present and retains GAP-activity but is rendered non-functional due to its mislocalization to the cytosol, while lentiviral shRNA-mediated knockdown in the human system leads to loss of more than 80% of RASA3. The *in vitro* model therefore offers a definitive examination of the erythroblast-intrinsic role of RASA3 but cannot recapitulate the intricacies of the phenotype caused by RASA3 mislocalization. The conditional mouse models, utilizing Cre-mediated excision of exon 3 of *RASA3* and resulting in the absence of RASA3 on the erythrocyte membrane, allow definitive examination of the cell-intrinsic role of RASA3 similar to the *in vitro* system, while also maintaining physiologic context and the hematopoietic niche. Of note, the absence of a phenotype in the *Epor*-*Cre* model indicates that RASA3 produced in early mouse erythroid progenitors can sustain terminal differentiation. However, in the human system, the RASA3 produced before lentiviral transduction was not sufficient to maintain normal terminal erythroid differentiation, indicating potential differences in RASA3 translation, stability, and requirements in human versus mouse terminal erythroid differentiation. Much future work will be required in order to fully characterize the specific mechanisms by which RASA3 regulates all aspects of mammalian hematopoiesis, including erythroid differentiation, to identify a potential new targetable axis in bone marrow failure syndromes.

## Methods

### Ethics statement

All experiments were performed in accordance with National Institutes of Health Laboratory Animal Care Guidelines and were approved by the Animal Care and Use Committee (ACUC) of The Jackson Laboratory (Protocol # 11006).

### Mice

All mice were maintained in climate- and light cycle-controlled animal facilities at The Jackson Laboratory. Acidified water and 5K52 chow (PMI LabDiet) were provided *ad libitum*. The spontaneous co-isogenic *scat* mutation arose originally on the inbred BALB/cBy substrain. Subsequently, *scat* was outbred with the BALB/cByJ substrain for three generations and then propagated by brother-sister mating in the research colonies of Dr. Luanne L. Peters at The Jackson Laboratory. Hence, the official designation of the *scat* mutant strain is CByJ;CByRasa3^*scat*^/LLP. All mouse strains used in this study are given in S9 Table.

### Generation of a *Rasa3* Conditional knockout (cKO) alleles

We generated a conditional targeted allele for *Rasa3* on a hybrid B6;129 genetic background using the *Rasa3*^tm1a(KOMP)Wtsi^ Knockout-first, promoter- driven conditional ready construct in which exon 3 is flanked by loxP sites (Fig 2A) that was generated by the trans-NIH Knock-Out Mouse Project (KOMP) and obtained from the KOMP Repository (www.komp.org). Targeted mice using the same construct were subsequently generated by the Jackson Laboratory KOMP Production Center on the inbred C57BL/6NJ (B6NJ) genetic background [53]. We electroporated the construct into 129S1/SvImJ (JAX Stock# 002448)-derived ES cells and cultured with G418 selection using standard protocols. Correctly targeted ES cells were identified by Southern blotting of genomic DNA digested with *Bcl1* and *Bgl1* using 5- and 3-prime flanking probes (Fig 2B) generated by PCR (primer sequences and expected product sizes provided in S10 Table). Blastocyst (C57BL/6J) injection and embryo transfer were performed using standard techniques. Heterozygous *Rasa3*^*cKO/+*^ offspring of male chimeras mated to C57BL/6J (B6J) females were identified by PCR genotyping (Fig 2C) and mated to *ACT-FLPe* transgenic mice to excise the IRES:*lacZ* and *neo* cassettes to generate the floxed allele, *Rasa3*^*fl*^ (S1 Fig). *Rasa3*^*fl/+*^ mice were bred with *CMV-Cre* mice to generate mice carrying a germline null allele (*Rasa3*^*+/-*^) and subsequently backcrossed to B6J mice to remove *CMV-Cre* (Fig 2D). *Rasa3*^*fl/-*^ and *Rasa3*^*fl/fl*^ mice were bred to Cre-expressing transgenic mice to generate cell type and tissue specific knockouts (S1 Fig).

### Identification of control and mutant mice

*Rasa3* and *Cre* genotypes in newborn progeny were determined initially using PCR (S10 Table) and subsequently by real time PCR with specific probes designed by Transnetyx. Mice designated as controls are Cre-negative fl/- and fl/fl and/or mice carrying a WT allele regardless of Cre genotype. Mutant mice are Cre-positive fl/- or fl/fl. Except where otherwise noted, all studies were performed using mice on the hybrid B6;129 genetic background.

### Polyinosinic-polycytidylic (Poly(I:C)) treatment

Floxed *Rasa3* exon 3 was excised in adult (8–12 weeks of age) hematopoietic cells by inducing *Mx1-Cre* recombinase activity with Poly(I:C) (GE Healthcare). Mice carrying floxed *Rasa3* allele(s) with and without (controls) *Mx1-Cre* were injected intraperitoneally with 300 μg of Poly(I:C) every other day for a total of five doses [54].

### Flow cytometric analysis of HSPC and terminal erythropoiesis

Dissociated whole spleens and bone marrow cells were passed through 100 μM Nitex nylon mesh (Genesee Scientific Products) with phosphate-buffered saline containing 0.5% bovine serum albumin and 2 mM EDTA (PBS/BSA/EDTA). Cell suspensions were filtered using CellTrics 50 μM disposable filters (Sysmex America), centrifuged at 300 x g for 10 minutes at 4°C, and resuspended in 3–4 mL of PBS/BSA/EDTA. Quantitative flow cytometric analysis of hematopoietic stem and progenitor cells (HSPCs) was performed as described [55] following staining with conjugated antibodies. Stem and myeloid (SMP) progenitor cells (Lin^-^Sca^-^Kit^+^CD34^+^CD16/32^lo^) cells from mice on the BALB genetic background were sorted as described [36]. For analysis of terminally differentiated erythroid precursors [28], single-cell suspensions prepared as above were depleted of CD45 positive cells using mouse CD45 microbeads (Miltenyi Biotec) prior to staining. Antibodies used in flow cytometric analyses are given in S11 Table. Stained cells were analyzed using LSR II Analyzer or FACSAria Sorter II (BD Biosciences) flow cytometers and FlowJo v. 9.9.3 software.

### Flow cytometric isolation of CD45+, Ter119+, and Ter119- cells from bone marrow and spleen

Single cell suspensions of whole spleen or bone marrow were prepared as above. CD45 positive cells were separated using mouse CD45 microbeads (Miltenyi Biotec). Ter119 positive cells were then isolated from the CD45 negative fraction using mouse Ter119 microbeads (Miltenyi Biotec) and magnetically separated using LS columns according to the manufacturer’s protocol. Ter119 negative cells were obtained as the flow-through fraction. Cell counts were performed using a Countess II Automated Cell Counter (Life Technologies) and/or Attune NXT Acoustic Focusing Cytometer (Thermo Fisher). Fractionated cell populations were used in western blotting (described below) or standard PCR reactions to genotype DNA for the presence of wildtype, floxed and/or excised alleles (S2 Table).

### In vitro colony-forming assays

Identification of BFU-E and CFU-E formation from whole bone marrow and spleen single cell suspensions was performed as described [56].

### Quantitative real-time RT-PCR (qRT-PCR) of HSPCs

Hematopoietic stem cells and progenitors including an enriched population of mixed erythroid BFU-E and CFU-E were isolated by flow cytometric cell sorting as described [19]. Total RNA was prepared using the PureLink RNA Mini Kit (Invitrogen). cDNA was synthesized using the High-Capacity cDNA Reverse Transcription Kit (Applied Biosystems) and random decamer primers (Invitrogen). qRT-PCR reactions (25 μL total volume) consisted of 10 μL of cDNA, 1.25ul dH2O, 12.5 μL of TaqMan Gene Expression Master Mix and 1.25 μL of primers and probes (TaqMan gene expression assay, assay ID: *Rasa3*, MM00436272.M1; Applied Biosystems). Samples were prepared in triplicate. qRT-PCR was performed using a ViiA7 Real-Time PCR System (Applied Biosystems) (40 cycles at 95°C for 15 sec and 60°C for 1 min). The relative quantification of each gene was determined by the 2^−ΔΔCT^ method using control CT values and *Gapdh* (TaqMan gene expression assay ID: Mn99999915_g1) expression for normalization.

### Peripheral blood analysis

Whole blood (250 μL) was collected via the retro-orbital sinus using dipotassium ethylenediaminetetraacetic acid (K_2_EDTA)-coated capillary tubes (Drummond Scientific) into BD Microtainer K_2_EDTA coated tubes (Becton, Dickinson and Company). Complete blood counts (CBCs) were obtained using an automated hematology analyzer equipped with species specific software (Advia 120 Multispecies Hematology Analyzer, Siemens Healthineers). Peripheral blood smears were stained with modified Wright-Giemsa (Sigma-Aldrich).

For cytokine measurements in bone marrow fluid, samples were prepared as described [57] with minor modifications. Briefly, bone marrow was flushed from femurs (0.5 cm) with 500 μL PBS using a 27 gauge syringe, resuspended, and centrifuged (600g, 5 min) to remove cells. Total protein content in bone marrow fluid was quantified by the method of Bradford [58] (Pierce kit, ThermoFisher Scientific). Bone marrow fluid samples and serum samples were analyzed by the University of Maryland Cytokine Core Laboratory Elisa Services (http://cytokines.com/).

### Phlebotomy

Adult *Mx1-Cre* negative control mice were rendered anemic by daily collection of ~350 μL whole blood via the retro-orbital sinus followed by intraperitoneal injections of sterile normal saline (37°C) for four days. Complete blood counts were obtained on day five.

### Sodium dodecyl sulfate–polyacrylamide gel electrophoresis (SDS-PAGE), western blotting and active RAS pull-down assays

Preparation of hemoglobin-depleted peripheral red blood cell membrane ghosts and whole cell lysates, SDS-PAGE and western blotting were performed as previously described [13]. Membrane ghost preparations were performed using packed red cells after removal of the buffy coat and thus are composed of both mature red cells and reticulocytes. Purified mature red cell and reticulocyte fractions for western blotting and RAS-GTP pull down assays were obtained as described [13]. Briefly, reticulocytes were separated from mature red cells by positive selection using antirat microbeads (Myltenyi) according to manufacturer instructions following staining with rat antimouse CD71 (Abcam).

The c-terminal peptide RASA3 primary antibody was generated as previously described [13]. GTP bound RAS was detected using the Active RAS Pull-Down and Detection Kit (Thermo Scientific) according to the manufacturer’s directions.

### Histology

Tissues were fixed in Bouin’s fixative, embedded in paraffin, sectioned at 3 μM and stained with hematoxylin and eosin for routine pathological analysis.

### RNAseq

Total RNA was isolated using the PureLink RNA Mini Kit (Invitrogen) and libraries prepared using the KAPA mRNA HyperPrep Kit (KAPA Biosystems) according to the manufacturer’s instructions, as previously described [20]. Libraries were checked for quality and concentration using the D5000 ScreenTape assay (Agilent Technologies) and quantitative PCR (KAPA Biosystems), according to the manufacturer’s instructions. Single-end sequencing was performed on the HiSeq2500 System using TruSeq SBS Kit v4 reagents (Illumina). Sequence reads were aligned and quantified with the Jackson Laboratory’s Computer Science group’s Civet Single-end RNAseq Analysis Pipeline (https://github.com/TheJacksonLaboratory/civet/). Briefly, sequences were preprocessed with an in-house python script that filtered sequences with less than seventy percent of the bases passing a quality threshold of 30, trimmed end bases with quality score less than 30, and removed sequences where the remaining length was less than seventy percent of the starting length. The resulting reads were mapped to mouse transcriptome (ENSEMBL version GRCm38.84) using bowtie *v2*.*2*.*0* (default parameters) and subsequent expression estimates were obtained using *rsem-calculate-expression v1*.*2*.*19* (default parameters). Upper quartile normalization of the resulting gene-level counts was accomplished with an in-house script. Count matrix normalization (rlog transformation) and differential expression analysis was performed with DESeq2 [59]. Differential expression analysis was performed on unnormalized counts assigned to each gene rather than FPKM (Fragments Per Kilobase of gene per Million reads), as recommended for DESeq2. Differentially expressed genes were extracted from DESeq2 output files using threshold values of ≥2 absolute fold change and ≤0.05 adjusted p-value.

To examine global patterns, further analysis was performed on the rlog regularized matrix. The regularized matrix was Z-transformed by the function *Z* = (*exp* − *avg*)/*stdev*, where *avg* and *stdev* are the average and sample standard deviation of the regularized expression level of each gene across all samples. To focus the analysis on genes with appreciable expression and/or variation, the matrix was reduced from all 38,925 annotated genes to 12,029 with *avg* ≥ 6.0 or *stdev* ≥ 2.0. Similar reductions were made on tissue or cell-type specific Z-transformed matrixes for more specific Principal Components Analysis.

Principal Components Analysis was performed using default parameters with JMP v11.2.1 (SAS Institute) on the transpose of the reduced Z-transformed expression matrixes. Gene ontology (GO) term searches were performed with DAVID v6.8 [38]. In some instances, highly redundant and higher order terms were removed in order to highlight additional processes.

### Lentivirus production

Lentivirus was produced by calcium chloride transfection of HEK-293T cells with 6.5μg pCMV-dR8.2 dvpr (Addgene #8455), 3.5μg pMD2.G (Addgene #12259), and 10μg pLKO.1-puro shRNA plasmids containing a puromycin resistance gene and the shRNA constructs of interest targeting *RASA3* (Sigma TRCN0000001639,40) or *Luciferase* (Sigma SHC007) per 10cm dish. sh*RASA3-39*, sh*RASA3-40*, and sh*Luciferase* sequences are CCGGACCTGAAGTTTGGAGATGAATCTCGAGATTCATCTCCAAACTTCAGGTTTTTT, CCGGAGCAAAGAAGACGAAAGTGAACTCGAGTTCACTTTCGTCTTCTTTGCTTTTTT, and CCGGCGCTGAGTACTTCGAAATGTCCTCGAGGACATTTCGAAGTACTCAGCGTTTTT, respectively. Transfection efficiency was verified by GFP control plasmid transfection. Viral supernatants were harvested at 48 and 72 hours post-transfection, 0.2μm filtered, and concentrated by ultracentrifugation at 20,000 rpm for 2 hours at 20°C. Concentrated virus was titrated by serial transduction of 3T3 cells followed by qPCR analysis.

### Lentiviral transduction

On day 2 of culture, cells were placed in transduction media comprised of IMDM, 10%FBS, 3U/mL heparin, and 8ug/mL polybrene and transduced with lentivirus containing sh*RASA3-39*,*40* or sh*Luciferase* at a multiplicity of infection (MOI) of 30 by centrifugal inoculation at 3000rpm for 2 hours. 12 hours post-transduction, media was changed to phase I media, and 48 hours post-transduction, puromycin selection with 1ug/mL puromycin was initiated, with puromycin maintained in culture through day 11. Successful knockdown was later verified by western blot.

### CD34+ cell cultures and flow cytometry to monitor terminal differentiation

Human CD34^+^ HSPCs were isolated from de-identified cord blood in accordance with Institutional Review Board approval (Northwell Health). CD34^+^ HSPCs were differentiated to enucleated reticulocytes using a 3-phase erythroid differentiation system as described previously [40]. Differentiation was assessed at days 8, 11, and 16 of culture. 10^5^ cells were stained with anti-GPA PE-conjugated (clone GAR-HIR2), anti-α4-integrin APC-conjugated (Clone REA545), and anti-band 3 FITC-conjugated (produced by Dr. Mohandas Narla). Erythroblasts were defined as 7AAD^neg^ GPA^pos^ cells, and the extent of terminal differentiation was measured by the surface expression of α4-integrin and band 3 [41].

### Image acquisition and assembly

Whole embryo images were acquired using a Nikon SMZ1500 stereomicroscope. Images of peripheral blood and tissue sections were acquired using a Nikon Eclipse E600 microscope equipped with a Nikon DS-Fi3 microscope camera and NIS-Elements Documentation software (Nikon Instruments). Embryos were examined with a 0.5X objective; smears with a 100X/1.4 oil-immersion objective; and tissue sections with a 20X/0.60 or 40X/0.75 objectives. Agarose gel thermal images and western blot films were scanned using a HP Scanjet G4010 scanner and HP Easy Scan software v.1.8 (Hewlett Packard). Final images were assembled using Microsoft PowerPoint for Mac v.16 (Microsoft).

### Statistical analysis

Significant differences were identified using Tukey honestly significant differences (HSD) or two-tailed Student *t* test using JMP v. 11 software (SAS Institute). Data underlying all graphs, tables, and summary statistics are given in S2 and S3 Data.

## Supporting information

S1 Fig Breeding strategy to generate *Rasa3* alleles. Schematic (adapted from Skarnes *et al*.)[53] showing strategy used to generate various *Rasa3* alleles. Targeted mutation (tm) designations used by convention by the KOMP (e.g., tm1a, tm1b, tm1c, and tm1d for the conditional ready targeted, germline null, floxed, and conditional null alleles, respectively) are indicated in blue. cKO, conditional knockout.
(DOCX)

S2 Fig Deletion of floxed alleles by *Epor-Cre* does not result in anemia. **(A)** Genotyping in Cd45^-^ Ter119^+^ spleen red cell precursors confirms excision of *Rasa3* floxed alleles in the presence of *Epor*-*Cre*. **(B)** Spleen weights as percent body weight (% BW). n = 3 per group. ns, not significantly different (p = 0.78). **(C)** Peripheral blood morphology is normal. A *scat* homozygote is shown for comparison. Bar, 10 μM. **(D)** Western blot of red cell membranes showing persistence of RASA3 protein in mutant mice. NS, non-specific band.
(DOCX)

S3 Fig Germline RASA3 null mice are embryonic lethal. Mice carrying the *Rasa3*^tm1a(KOMP)Wtsi)^ allele were produced on the inbred C57BL/6NJ (B6NJ) background by the KOMP at The Jackson Laboratory [60]. Germline homozygous null mice generated by breeding with *Sox2-Cre* expressing transgenic mice (B6N.Cg-*Edil3*^*Tg(Sox2-cre)1Amc*^/J) die at E12.5-E13.5 with a phenotype of severe hemorrhage, overall pallor and a small, pale fetal liver (right, arrow). Additional data and images are available at the International Mouse Phenotyping Consortium (IMPC) website (www.mousephenotype.org).
(DOCX)

S4 Fig Frequency of hematopoietic stem and progenitors. **(A)** Bone Marrow. **(B)** Spleen. LT-HSC, long term hematopoietic stem cell; ST-HSC, short term hematopoietic stem cell; LMPP, lymphoid-primed multipotent progenitor; CLP, common lymphoid progenitor; CMP, common myeloid progenitor; GMP, granulocyte monocyte progenitor; MEP, Myeloid-erythroid progenitor. n = 8 per group. * p < = 05.
(DOCX)

S5 Fig Spleen histology. **(A)** *Mx1-Cre*; *Rasa3* control and **(B)** mutant spleen. Effacement of the normal splenic nodular architecture and increased megakaryocytes are evident in the mutant spleen within 2 weeks of pIpC treatment. Original magnification 100x.
(PDF)

S6 Fig Spleen histology. **(A)** B6J-+/+ **(B)** B6J-*hlb381/hlb381*. Original magnification 100x.
(DOCX)

S7 Fig Analysis of global expression in bone marrow. Hierarchal clustering **(A)** and principle component analysis **(B)** of expression differences in *scat vs*. WT bone marrow.
(DOCX)

S8 Fig Functional gene ontology (GO) terms for differentially expressed genes in spleen and bone marrow. Data for all mutants (combined cr and pr) vs. WT in **(A)** spleen and (**B)** bone marrow. David BP_FAT function.
(DOCX)

S9 Fig Functional gene ontology (GO) terms for differentially expressed genes in spleen and bone marrow SMP (stem and myeloid progenitors) cells. Data for all mutants (combined cr and pr) vs. WT in **(A)** spleen SMP cells and (**B)** bone marrow SMP cells. David BP_FAT function.
(DOCX)

S10 Fig Functional gene ontology (GO) terms for differentially expressed genes in spleen and bone marrow MEP (megakaryocyte-erythroid progenitors) cells. Data for all mutants (combined cr and pr) vs. WT in **(A)** spleen MEP cells and (**B)** bone marrow MEP cells. David BP_FAT function.
(DOCX)

S11 Fig Confirmation of impaired human terminal erythropoiesis by RASA3 knockdown using a different shRNA construct. **(A)** Verification of RASA3 knockdown by a second shRNA construct. **(B)** Delay in terminal erythropoiesis along with **(C)** respective quantifications at Day 11 and 16 (n = 4 and 3, respectively).
(DOCX)

S12 Fig RASA3 knockdown does not impair proliferation of CD34+ cells. Proliferation between days 7 and 11 in vitro, demonstrating no differences between sh*Luciferase* and shRASA3-39 cultures (n = 4).
(DOCX)

S1 Table Complete blood counts in *Epor-Cre; Rasa3* adult mice 6–8 weeks of age. (DOCX)

S2 Table Complete blood counts in *scat* mice 3–5 weeks of age. (DOCX)

S3 Table Complete blood counts in phlebotomized (PHB) control vs. mutant mice. (DOCX)

S4 Table Complete blood counts in B6NJ *Mx1-Cre; Rasa3* adult mice. (DOCX)

S5 Table Complete blood counts in C57BL/6J-*hlb381* mice 3–4 weeks of age. (DOCX)

S6 Table Complete blood counts in C57BL/6J-*hlb381* mice ≥ 6 weeks of age. (DOCX)

S7 Table RNAseq biological replicates. (DOCX)

S8 Table Complete blood counts in *scat* mice used for RNAseq. (DOCX)

S9 Table Mouse strains. (DOCX)

S10 Table Genotyping primers and product sizes. (DOCX)

S11 Table Flow cytometry antibodies. (DOCX)

S1 Data RNAseq datasets. (XLSX)

S2 Data Data underlying all figures and tables (mouse studies). (XLSX)

S3 Data Data underlying studies in CD34+ cells. (XLSX)

## References

[1] Yarwood S, Bouyoucef-Cherchalli D, Cullen PJ, Kupzig S. The GAP1 family of GTPase-activating proteins: spatial and temporal regulators of small GTPase signalling. Biochem Soc Trans. 2006;34(Pt 5):846–50. Epub 2006/10/21. 10.1042/BST0340846 . 17052212

[2] Fukuda M, Mikoshiba K. Structure-function relationships of the mouse Gap1m. Determination of the inositol 1,3,4,5-tetrakisphosphate-binding domain. J Biol Chem. 1996;271(31):18838–42. Epub 1996/08/02. 10.1074/jbc.271.31.18838 . 8702543

[3] King PD, Lubeck BA, Lapinski PE. Nonredundant functions for Ras GTPase-activating proteins in tissue homeostasis. Sci Signal. 2013;6(264):re1 Epub 2013/02/28. 10.1126/scisignal.2003669 23443682 PMC5483993

[4] Kupzig S, Deaconescu D, Bouyoucef D, Walker SA, Liu Q, Polte CL, et al GAP1 family members constitute bifunctional Ras and Rap GTPase-activating proteins. J Biol Chem. 2006;281(15):9891–900. Epub 2006/01/25. 10.1074/jbc.M512802200 16431904 PMC1904491

[5] Sot B, Behrmann E, Raunser S, Wittinghofer A. Ras GTPase activating (RasGAP) activity of the dual specificity GAP protein Rasal requires colocalization and C2 domain binding to lipid membranes. Proc Natl Acad Sci U S A. 2013;110(1):111–6. Epub 2012/12/20. 10.1073/pnas.1201658110 23251034 PMC3538246

[6] Sot B, Kotting C, Deaconescu D, Suveyzdis Y, Gerwert K, Wittinghofer A. Unravelling the mechanism of dual-specificity GAPs. EMBO J. 2010;29(7):1205–14. Epub 2010/02/27. 10.1038/emboj.2010.20 20186121 PMC2857463

[7] Khalaf WF, White H, Wenning MJ, Orazi A, Kapur R, Ingram DA. K-Ras is essential for normal fetal liver erythropoiesis. Blood. 2005;105(9):3538–41. Epub 2005/01/13. 10.1182/blood-2004-05-2021 . 15644420

[8] Zhang J, Liu Y, Beard C, Tuveson DA, Jaenisch R, Jacks TE, et al Expression of oncogenic K-ras from its endogenous promoter leads to a partial block of erythroid differentiation and hyperactivation of cytokine-dependent signaling pathways. Blood. 2007;109(12):5238–41. Epub 2007/02/24. 10.1182/blood-2006-09-047050 17317860 PMC1890843

[9] Zhang J, Lodish HF. Endogenous K-ras signaling in erythroid differentiation. Cell cycle. 2007;6(16):1970–3. Epub 2007/08/28. 10.4161/cc.6.16.4577 . 17721087

[10] Kaushansky K. The molecular mechanisms that control thrombopoiesis. J Clin Invest. 2005;115(12):3339–47. Epub 2005/12/03. 10.1172/JCI26674 16322778 PMC1297257

[11] Frische EW, Zwartkruis FJ. Rap1, a mercenary among the Ras-like GTPases. Dev Biol. 2010;340(1):1–9. Epub 2010/01/12. 10.1016/j.ydbio.2009.12.043 . 20060392

[12] Iwashita S, Kobayashi M, Kubo Y, Hinohara Y, Sezaki M, Nakamura K, et al Versatile roles of R-Ras GAP in neurite formation of PC12 cells and embryonic vascular development. The Journal of biological chemistry. 2007;282(6):3413–7. Epub 2006/12/21. 10.1074/jbc.C600293200 . 17179160

[13] Blanc L, Ciciotte SL, Gwynn B, Hildick-Smith GJ, Pierce EL, Soltis KA, et al Critical function for the Ras-GTPase activating protein RASA3 in vertebrate erythropoiesis and megakaryopoiesis. Proc Natl Acad Sci U S A. 2012;109(30):12099–104. Epub 2012/07/10. 10.1073/pnas.1204948109 22773809 PMC3409772

[14] Peters LL, McFarland-Starr EC, Wood BG, Barker JE. Heritable severe combined anemia and thrombocytopenia in the mouse: description of the disease and successful therapy. Blood. 1990;76(4):745–54. Epub 1990/08/15. . 2383655

[15] Stefanini L, Paul DS, Robledo RF, Chan ER, Getz TM, Campbell RA, et al RASA3 is a critical inhibitor of RAP1-dependent platelet activation. J Clin Invest. 2015;125(4):1419–32. Epub 2015/02/24. 10.1172/JCI77993 25705885 PMC4396462

[16] Molina-Ortiz P, Orban T, Martin M, Habets A, Dequiedt F, Schurmans S. Rasa3 controls turnover of endothelial cell adhesion and vascular lumen integrity by a Rap1-dependent mechanism. PLoS Genet. 2018;14(1):e1007195 Epub 2018/01/31. 10.1371/journal.pgen.1007195 29381707 PMC5806903

[17] Molina-Ortiz P, Polizzi S, Ramery E, Gayral S, Delierneux C, Oury C, et al Rasa3 controls megakaryocyte Rap1 activation, integrin signaling and differentiation into proplatelet. PLoS Genet. 2014;10(6):e1004420 Epub 2014/06/27. 10.1371/journal.pgen.1004420 24967784 PMC4072513

[18] Heinrich AC, Pelanda R, Klingmuller U. A mouse model for visualization and conditional mutations in the erythroid lineage. Blood. 2004;104(3):659–66. Epub 2004/04/20. 10.1182/blood-2003-05-1442 . 15090451

[19] Flygare J, Rayon Estrada V, Shin C, Gupta S, Lodish HF. HIF1alpha synergizes with glucocorticoids to promote BFU-E progenitor self-renewal. Blood. 2011;117(12):3435–44. Epub 2010/12/24. 10.1182/blood-2010-07-295550 21177435 PMC3069680

[20] Nebor D, Graber JH, Ciciotte SL, Robledo RF, Papoin J, Hartman E, et al Mutant KLF1 in Adult Anemic Nan Mice Leads to Profound Transcriptome Changes and Disordered Erythropoiesis. Sci Rep. 2018;8(1):12793 Epub 2018/08/26. 10.1038/s41598-018-30839-2 30143664 PMC6109071

[21] Tusi BK, Wolock SL, Weinreb C, Hwang Y, Hidalgo D, Zilionis R, et al Population snapshots predict early haematopoietic and erythroid hierarchies. Nature. 2018;555(7694):54–60. Epub 2018/02/22. 10.1038/nature25741 29466336 PMC5899604

[22] de Boer J, Williams A, Skavdis G, Harker N, Coles M, Tolaini M, et al Transgenic mice with hematopoietic and lymphoid specific expression of Cre. Eur J Immunol. 2003;33(2):314–25. Epub 2003/01/28. 10.1002/immu.200310005 . 12548562

[23] Joseph C, Quach JM, Walkley CR, Lane SW, Lo Celso C, Purton LE. Deciphering hematopoietic stem cells in their niches: a critical appraisal of genetic models, lineage tracing, and imaging strategies. Cell Stem Cell. 2013;13(5):520–33. Epub 2013/11/12. 10.1016/j.stem.2013.10.010 . 24209759

[24] Ding L, Saunders TL, Enikolopov G, Morrison SJ. Endothelial and perivascular cells maintain haematopoietic stem cells. Nature. 2012;481(7382):457–62. Epub 2012/01/28. 10.1038/nature10783 22281595 PMC3270376

[25] Hartner JC, Walkley CR, Lu J, Orkin SH. ADAR1 is essential for the maintenance of hematopoiesis and suppression of interferon signaling. Nat Immunol. 2009;10(1):109–15. Epub 2008/12/09. 10.1038/ni.1680 19060901 PMC2701568

[26] Kuhn R, Schwenk F, Aguet M, Rajewsky K. Inducible gene targeting in mice. Science. 1995;269(5229):1427–9. Epub 1995/09/08. 10.1126/science.7660125 . 7660125

[27] Hartman ES, Brindley EC, Papoin J, Ciciotte SL, Zhao Y, Peters LL, et al Increased Reactive Oxygen Species and Cell Cycle Defects Contribute to Anemia in the RASA3 Mutant Mouse Model scat. Front Physiol. 2018;9:689 Epub 2018/06/21. 10.3389/fphys.2018.00689 29922180 PMC5996270

[28] Liu J, Zhang J, Ginzburg Y, Li H, Xue F, De Franceschi L, et al Quantitative analysis of murine terminal erythroid differentiation in vivo: novel method to study normal and disordered erythropoiesis. Blood. 2013;121(8):e43–9. Epub 2013/01/05. 10.1182/blood-2012-09-456079 23287863 PMC3578961

[29] Paulson RF, Shi L, Wu DC. Stress erythropoiesis: new signals and new stress progenitor cells. Curr Opin Hematol. 2011;18(3):139–45. Epub 2011/03/05. 10.1097/MOH.0b013e32834521c8 21372709 PMC3099455

[30] Trompouki E, Bowman TV, Lawton LN, Fan ZP, Wu DC, DiBiase A, et al Lineage regulators direct BMP and Wnt pathways to cell-specific programs during differentiation and regeneration. Cell. 2011;147(3):577–89. Epub 2011/11/01. 10.1016/j.cell.2011.09.044 PubMed Central PMCID: PMC3219441. 22036566 PMC3219441

[31] Brecher G, Stohlman F Jr. Reticulocyte size and erythropoietic stimulation. Proc Soc Exp Biol Med. 1961;107:887–91. Epub 1961/08/01. 10.3181/00379727-107-26785 . 13872711

[32] Hillman RS. The importance of iron supply in thalassemic erythropoiesis. Ann N Y Acad Sci. 1969;165(1):100–4. Epub 1969/11/20. 10.1111/j.1749-6632.1969.tb27780.x . 5260135

[33] Hillman RS. Characteristics of marrow production and reticulocyte maturation in normal man in response to anemia. J Clin Invest. 1969;48(3):443–53. Epub 1969/03/01. 10.1172/JCI106001 5773082 PMC535708

[34] Clark AT, Goldowitz D, Takahashi JS, Vitaterna MH, Siepkas SM, Peters LL, et al Implementing large-scale ENU mutagenesis screens in North America. Genetica. 2004;122:51–64. 10.1007/s10709-004-1436-6 15619961 PMC3774779

[35] Blanc L, Ciciotte SL, Lipton JM, Liu JM, Peters LL. RASA3 plays a critical, conserved role in erythroid differentiation. Blood. 2012;120:3186.

[36] Spangrude GJ, Brooks DM. Mouse strain variability in the expression of the hematopoietic stem cell antigen Ly-6A/E by bone marrow cells. Blood. 1993;82(11):3327–32. Epub 1993/12/01. . 8241503

[37] Bumm TG, Elsea C, Corbin AS, Loriaux M, Sherbenou D, Wood L, et al Characterization of murine JAK2V617F-positive myeloproliferative disease. Cancer research. 2006;66(23):11156–65. 10.1158/0008-5472.CAN-06-2210 . 17145859

[38] DennisGJr., ShermanBT, HosackDA, YangJ, GaoW, LaneHC, et al DAVID: Database for Annotation, Visualization, and Integrated Discovery. Genome Biol. 2003;4(5):P3 Epub 2003/05/08. .12734009

[39] An interactive tool for comparing lists with Venn's diagrams. [*http://bioinfogp.cnb.csic.es/tools/venny/index.html* 2007–2015]. 2007–2015.

[40] Dulmovits BM, Appiah-Kubi AO, Papoin J, Hale J, He M, Al-Abed Y, et al Pomalidomide reverses gamma-globin silencing through the transcriptional reprogramming of adult hematopoietic progenitors. Blood. 2016;127(11):1481–92. Epub 2015/12/19. 10.1182/blood-2015-09-667923 26679864 PMC4797024

[41] Hu J, Liu J, Xue F, Halverson G, Reid M, Guo A, et al Isolation and functional characterization of human erythroblasts at distinct stages: implications for understanding of normal and disordered erythropoiesis in vivo. Blood. 2013;121(16):3246–53. Epub 2013/02/21. 10.1182/blood-2013-01-476390 23422750 PMC3630836

[42] Li QF, Decker-Rockefeller B, Bajaj A, Pumiglia K. Activation of Ras in the Vascular Endothelium Induces Brain Vascular Malformations and Hemorrhagic Stroke. Cell Rep. 2018;24(11):2869–82. Epub 2018/09/13. 10.1016/j.celrep.2018.08.025 . 30208313

[43] Palis J. Primitive and definitive erythropoiesis in mammals. Front Physiol. 2014;5:3 Epub 2014/01/31. 10.3389/fphys.2014.00003 24478716 PMC3904103

[44] Schurmans S, Polizzi S, Scoumanne A, Sayyed S, Molina-Ortiz P. The Ras/Rap GTPase activating protein RASA3: from gene structure to in vivo functions. Adv Biol Regul. 2015;57:153–61. Epub 2014/10/09. 10.1016/j.jbior.2014.09.006 . 25294679

[45] Zhang J, Lodish HF. Constitutive activation of the MEK/ERK pathway mediates all effects of oncogenic H-ras expression in primary erythroid progenitors. Blood. 2004;104(6):1679–87. Epub 2004/05/29. 10.1182/blood-2004-04-1362 . 15166036

[46] Jung M, Ramanagoudr-Bhojappa R, van Twest S, Rosti RO, Murphy V, Tan W, et al Association of clinical severity with FANCB variant type in Fanconi anemia. Blood. 2020;135(18):1588–602. Epub 2020/02/28. 10.1182/blood.2019003249 32106311 PMC7193183

[47] Vlachos A, Klein GW, Lipton JM. The Diamond Blackfan Anemia Registry: tool for investigating the epidemiology and biology of Diamond-Blackfan anemia. J Pediatr Hematol Oncol. 2001;23(6):377–82. Epub 2001/09/21. 10.1097/00043426-200108000-00015 . 11563775

[48] Rinehart J, Gulcicek EE, Joiner CH, Lifton RP, Gallagher PG. Determinants of erythrocyte hydration. Curr Opin Hematol. 2010;17(3):191–7. Epub 2010/02/26. 10.1097/MOH.0b013e32833800d0 20182354 PMC4155397

[49] Glogowska E, Lezon-Geyda K, Maksimova Y, Schulz VP, Gallagher PG. Mutations in the Gardos channel (KCNN4) are associated with hereditary xerocytosis. Blood. 2015;126(11):1281–4. Epub 2015/07/23. 10.1182/blood-2015-07-657957 26198474 PMC4566808

[50] Glogowska E, Schneider ER, Maksimova Y, Schulz VP, Lezon-Geyda K, Wu J, et al Novel mechanisms of PIEZO1 dysfunction in hereditary xerocytosis. Blood. 2017;130(16):1845–56. Epub 2017/07/19. 10.1182/blood-2017-05-786004 28716860 PMC5649553

[51] Shmukler BE, Rivera A, Bhargava P, Nishimura K, Kim EH, Hsu A, et al Genetic disruption of KCC cotransporters in a mouse model of thalassemia intermedia. Blood Cells Mol Dis. 2020;81:102389 Epub 2019/12/14. 10.1016/j.bcmd.2019.102389 31835175 PMC7002294

[52] Shmukler BE, Rivera A, Bhargava P, Nishimura K, Hsu A, Kim EH, et al Combined genetic disruption of K-Cl cotransporters and Gardos channel KCNN4 rescues erythrocyte dehydration in the SAD mouse model of sickle cell disease. Blood Cells Mol Dis. 2019;79:102346 Epub 2019/07/29. 10.1016/j.bcmd.2019.102346 31352162 PMC6744291

[53] Skarnes WC, Rosen B, West AP, Koutsourakis M, Bushell W, Iyer V, et al A conditional knockout resource for the genome-wide study of mouse gene function. Nature. 2011;474(7351):337–42. Epub 2011/06/17. 10.1038/nature10163 21677750 PMC3572410

[54] Kalfa TA, Pushkaran S, Mohandas N, Hartwig JH, Fowler VM, Johnson JF, et al Rac GTPases regulate the morphology and deformability of the erythrocyte cytoskeleton. Blood. 2006;108(12):3637–45. Epub 2006/08/03. 10.1182/blood-2006-03-005942 16882712 PMC1895472

[55] Challen GA, Boles N, Lin KK, Goodell MA. Mouse hematopoietic stem cell identification and analysis. Cytometry A. 2009;75(1):14–24. Epub 2008/11/22. 10.1002/cyto.a.20674 19023891 PMC2640229

[56] Kalfa TA, Pushkaran S, Zhang X, Johnson JF, Pan D, Daria D, et al Rac1 and Rac2 GTPases are necessary for early erythropoietic expansion in the bone marrow but not in the spleen. Haematologica. 2010;95(1):27–35. Epub 2010/01/13. 10.3324/haematol.2009.006239 20065081 PMC2805739

[57] Dar A, Schajnovitz A, Lapid K, Kalinkovich A, Itkin T, Ludin A, et al Rapid mobilization of hematopoietic progenitors by AMD3100 and catecholamines is mediated by CXCR4-dependent SDF-1 release from bone marrow stromal cells. Leukemia. 2011;25(8):1286–96. Epub 2011/04/16. 10.1038/leu.2011.62 21494253 PMC4175714

[58] Bradford MM. A rapid and sensitive method for the quantitation of microgram quantities of protein utilizing the principle of protein-dye binding. Anal Biochem. 1976;72:248–54. Epub 1976/05/07. 10.1006/abio.1976.9999 . 942051

[59] Love MI, Huber W, Anders S. Moderated estimation of fold change and dispersion for RNA-seq data with DESeq2. Genome Biol. 2014;15(12):550 Epub 2014/12/18. 10.1186/s13059-014-0550-8 25516281 PMC4302049

[60] Dickinson ME, Flenniken AM, Ji X, Teboul L, Wong MD, White JK, et al High-throughput discovery of novel developmental phenotypes. Nature. 2016;537(7621):508–14. Epub 2016/09/15. 10.1038/nature19356 27626380 PMC5295821

