# Supplementary material for: Differential effects of RASA3 mutations on hematopoiesis are profoundly influenced by genetic background and molecular variant
Source: PLoS Genet. 2020 Dec 28;16(12):e1008857. doi: 10.1371/journal.pgen.1008857 (PMC7793307; doi:10.1371/journal.pgen.1008857)
Supplement: S1 Fig — Schematic (adapted from Skarnes et al.)[53] showing strategy used to generate various Rasa3 alleles. Targeted mutation (tm) designations used by convention by the KOMP (e.g., tm1a, tm1b, tm1c, and tm1d for the conditional ready targeted, germline null, floxed, and conditional null alleles, respectively) are indicated in blue. cKO, conditional knockout. (DOCX) [file pgen.1008857.s001.docx]

**Reference**

1. Skarnes WC, Rosen B, West AP, Koutsourakis M, Bushell W, Iyer V, et al. A conditional knockout resource for the genome-wide study of mouse gene function. Nature. 2011;474(7351):337-42. Epub 2011/06/17. doi: 10.1038/nature10163. PubMed PMID: 21677750; PubMed Central PMCID: PMCPMC3572410.

**S1 Fig**
