# Supplementary material for: Differential effects of RASA3 mutations on hematopoiesis are profoundly influenced by genetic background and molecular variant
Source: PLoS Genet. 2020 Dec 28;16(12):e1008857. doi: 10.1371/journal.pgen.1008857 (PMC7793307; doi:10.1371/journal.pgen.1008857)
Supplement: S3 Fig — Mice carrying the Rasa3tm1a(KOMP)Wtsi) allele were produced on the inbred C57BL/6NJ (B6NJ) background by the KOMP at The Jackson Laboratory [60]. Germline homozygous null mice generated by breeding with Sox2-Cre expressing transgenic mice (B6N.Cg-Edil3Tg(Sox2-cre)1Amc/J) die at E12.5-E13.5 with a phenotype of severe hemorrhage, overall pallor and a small, pale fetal liver (right, arrow). Additional data and images are available at the International Mouse Phenotyping Consortium (IMPC) website (www.mousephenotype.org). (DOCX) [file pgen.1008857.s003.docx]

**Reference**

1. Dickinson ME, Flenniken AM, Ji X, Teboul L, Wong MD, White JK, et al. High-throughput discovery of novel developmental phenotypes. Nature. 2016;537(7621):508-14. Epub 2016/09/15. doi: 10.1038/nature19356. PubMed PMID: 27626380; PubMed Central PMCID: PMCPMC5295821.


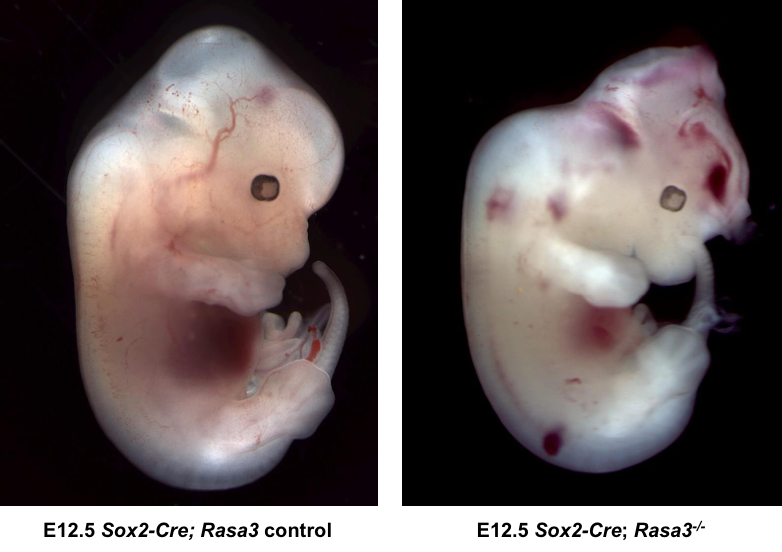


**→**

**→**

**S3 Fig**
