# Supplementary figures and images for: Differential effects of RASA3 mutations on hematopoiesis are profoundly influenced by genetic background and molecular variant
Source: PLoS Genet. 2020 Dec 28;16(12):e1008857. doi: 10.1371/journal.pgen.1008857 (PMC7793307; doi:10.1371/journal.pgen.1008857)

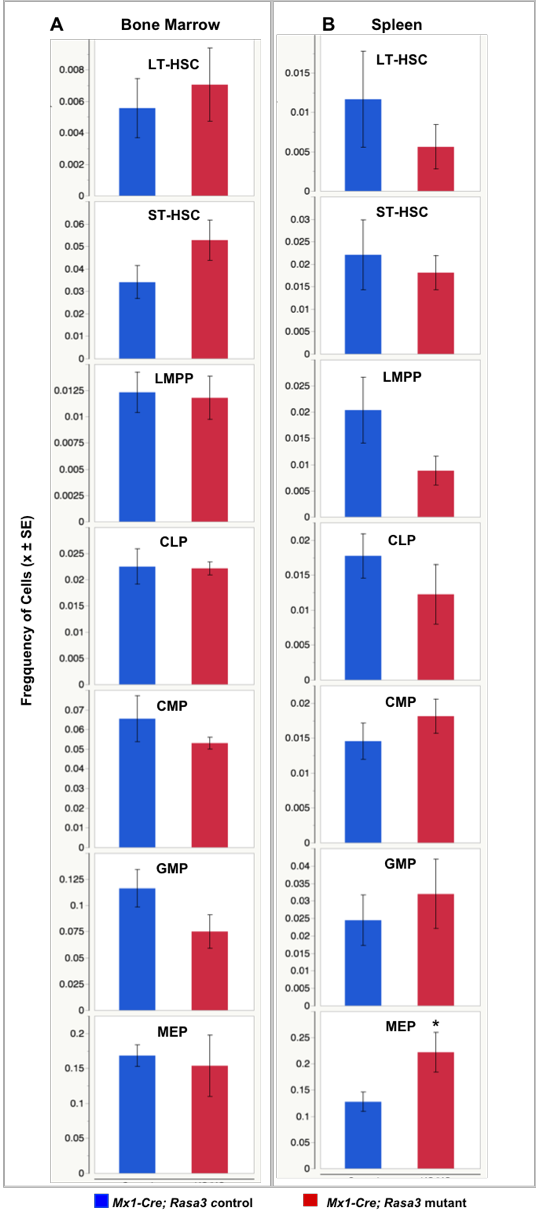


**S4 Fig**

**Frequency of Cells (X ± sem)**

**b**

**b**

Supplement: S4 Fig — (A) Bone Marrow. (B) Spleen. LT-HSC, long term hematopoietic stem cell; ST-HSC, short term hematopoietic stem cell; LMPP, lymphoid-primed multipotent progenitor; CLP, common lymphoid progenitor; CMP, common myeloid progenitor; GMP, granulocyte monocyte progenitor; MEP, Myeloid-erythroid progenitor. n = 8 per group. * p < = 05. (DOCX) [file pgen.1008857.s004.docx]

**S5 Fig**

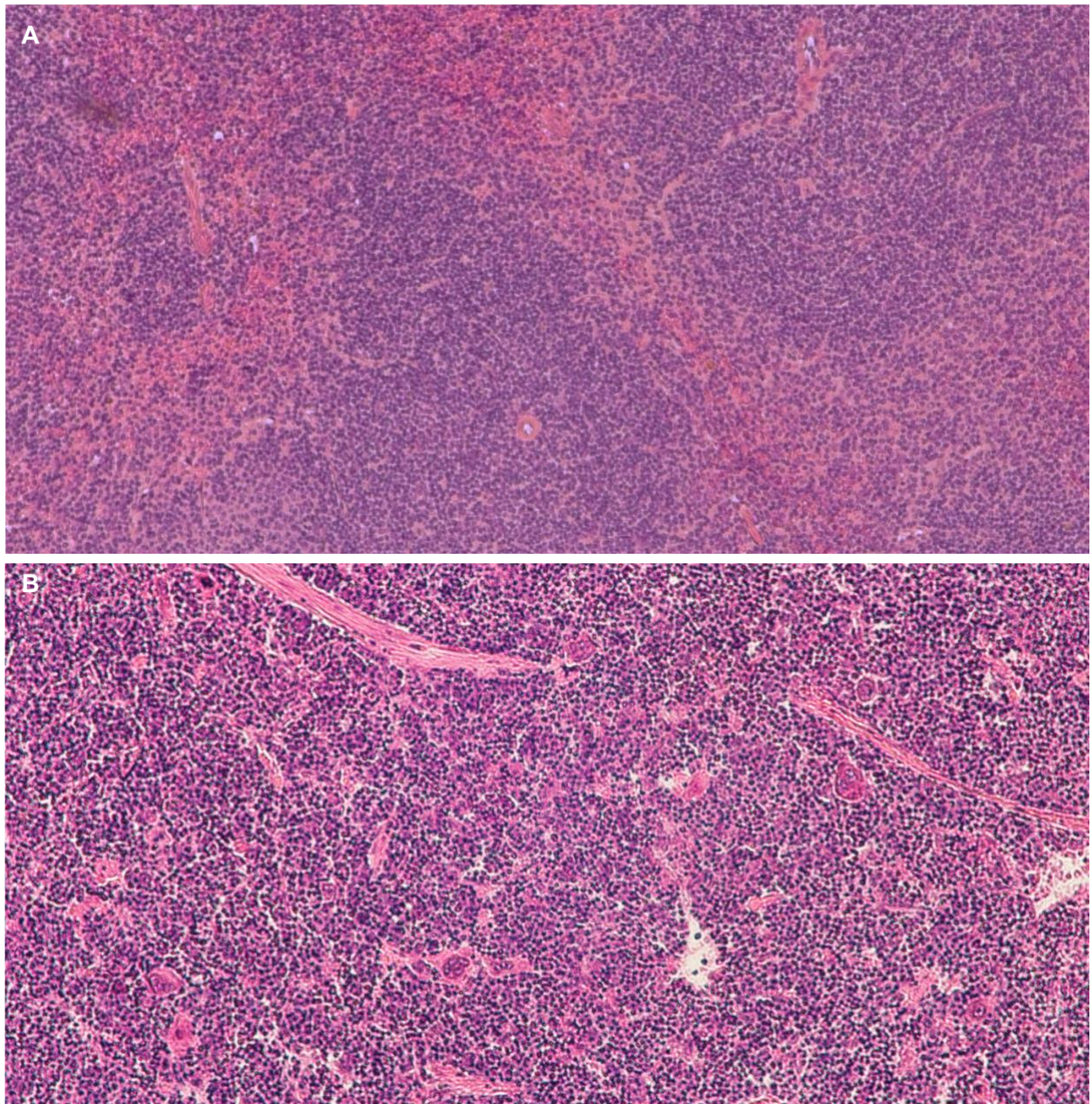

Supplement: S5 Fig — (A) Mx1-Cre; Rasa3 control and (B) mutant spleen. Effacement of the normal splenic nodular architecture and increased megakaryocytes are evident in the mutant spleen within 2 weeks of pIpC treatment. Original magnification 100x. (PDF) [file pgen.1008857.s005.pdf]

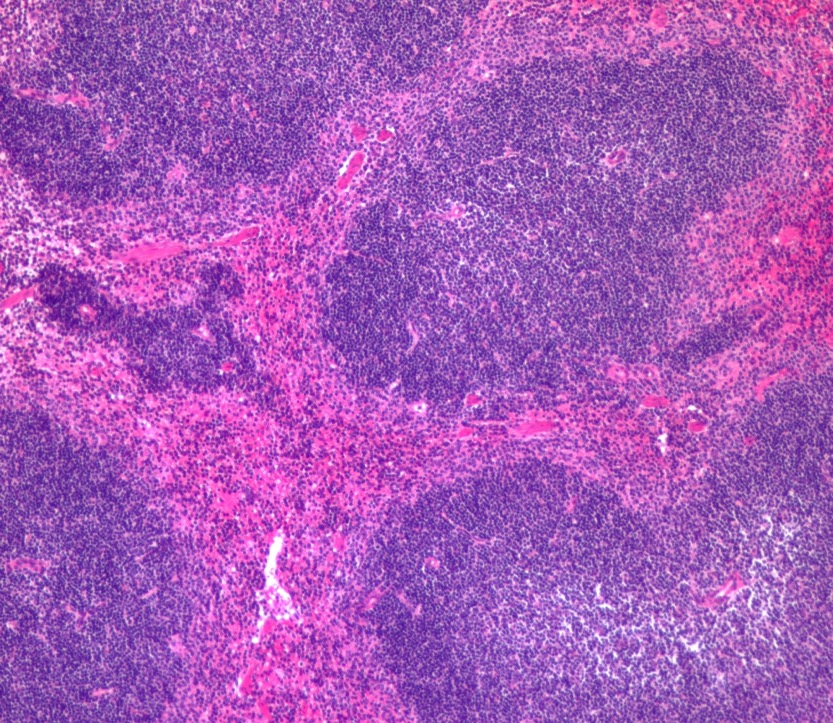

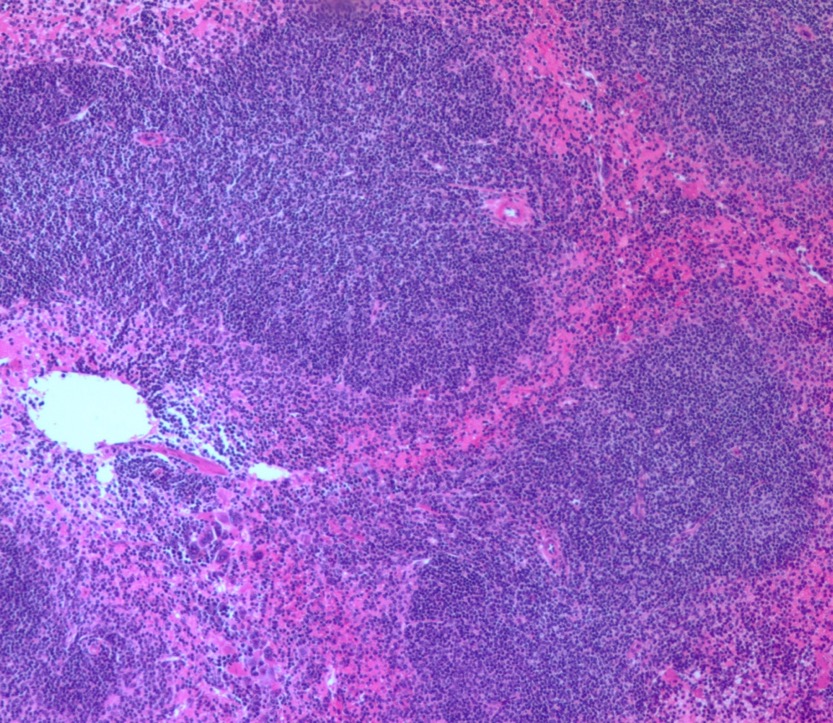


**A**

**B**

**S6 Fig**

hlb

B6

Supplement: S6 Fig — (A) B6J-+/+ (B) B6J-hlb381/hlb381. Original magnification 100x. (DOCX) [file pgen.1008857.s006.docx]

**S11 Fig**


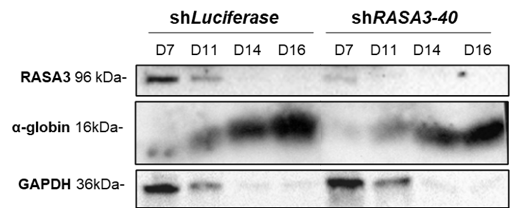

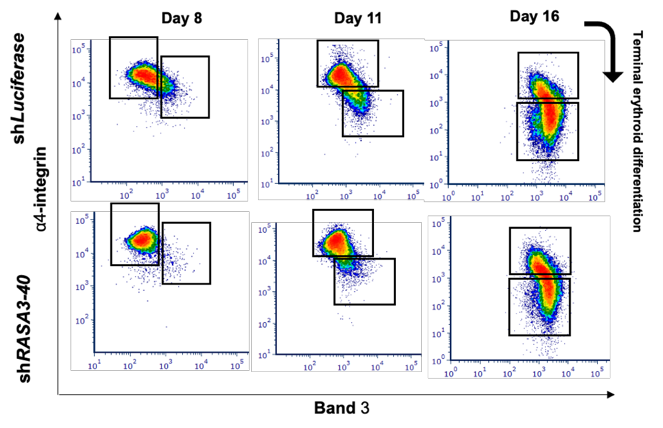

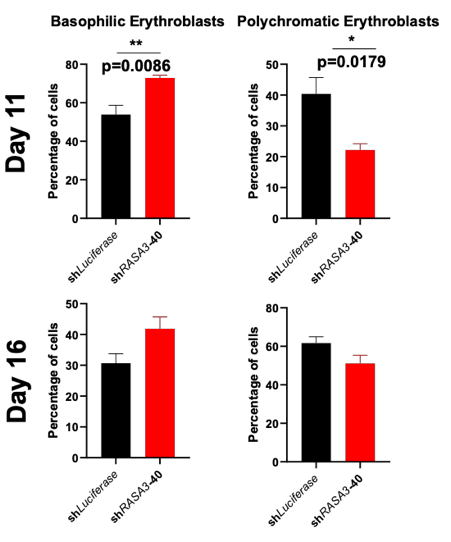


**C**

**A**

B

Supplement: S11 Fig — (A) Verification of RASA3 knockdown by a second shRNA construct. (B) Delay in terminal erythropoiesis along with (C) respective quantifications at Day 11 and 16 (n = 4 and 3, respectively). (DOCX) [file pgen.1008857.s011.docx]

**S12 Fig**


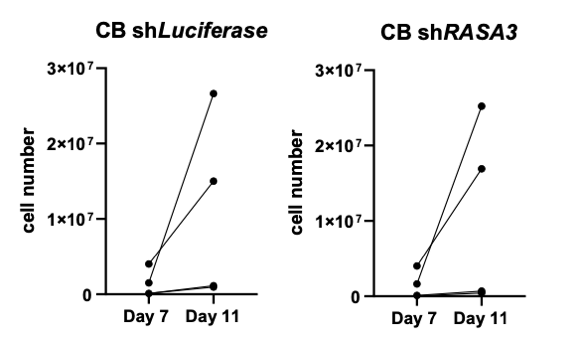

Supplement: S12 Fig — Proliferation between days 7 and 11 in vitro, demonstrating no differences between shLuciferase and shRASA3-39 cultures (n = 4). (DOCX) [file pgen.1008857.s012.docx]
