# Supplementary material for: Differential effects of RASA3 mutations on hematopoiesis are profoundly influenced by genetic background and molecular variant
Source: PLoS Genet. 2020 Dec 28;16(12):e1008857. doi: 10.1371/journal.pgen.1008857 (PMC7793307; doi:10.1371/journal.pgen.1008857)
Supplement: S7 Fig — Hierarchal clustering (A) and principle component analysis (B) of expression differences in scat vs. WT bone marrow. (DOCX) [file pgen.1008857.s007.docx]

hlb

B6

**B**

**A**

**Whole Bone**

**Marrow**

**SMP**

**MEP**


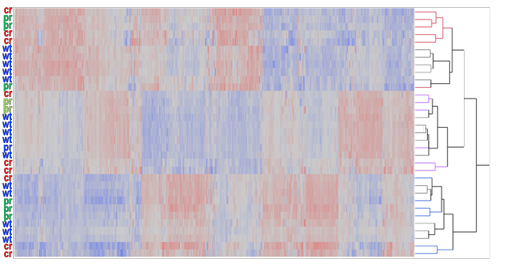


**WT *scat* cr *scat* pr**

**WT *scat* cr *scat*  pr**

**50**

**0**

**-50**

**100**

**PC2 (24.9 %)**

**PC1 (30.1 %)**

**-50 0 50 100**

**Whole Bone Marrow**

**50**

**0**

**-50**

**PC2 (16.7 %)**

**PC1 (34.8 %)**

**-150 -100 -50 0 50**

**Bone Marrow SMP**

**50**

**0**

**-50**

**100**

**PC2 (20.9 %)**

**PC1 (32.1 %)**

**-100 -50 0 50 100**

**Bone Marrow MEP**

**S7 Fig**
