# Supplementary material for: Differential effects of RASA3 mutations on hematopoiesis are profoundly influenced by genetic background and molecular variant
Source: PLoS Genet. 2020 Dec 28;16(12):e1008857. doi: 10.1371/journal.pgen.1008857 (PMC7793307; doi:10.1371/journal.pgen.1008857)
Supplement: S10 Fig — Data for all mutants (combined cr and pr) vs. WT in (A) spleen MEP cells and (B) bone marrow MEP cells. David BP_FAT function. (DOCX) [file pgen.1008857.s010.docx]

**S10 Fig**

**A MEP Spleen**

**B MEP Bone Marrow**


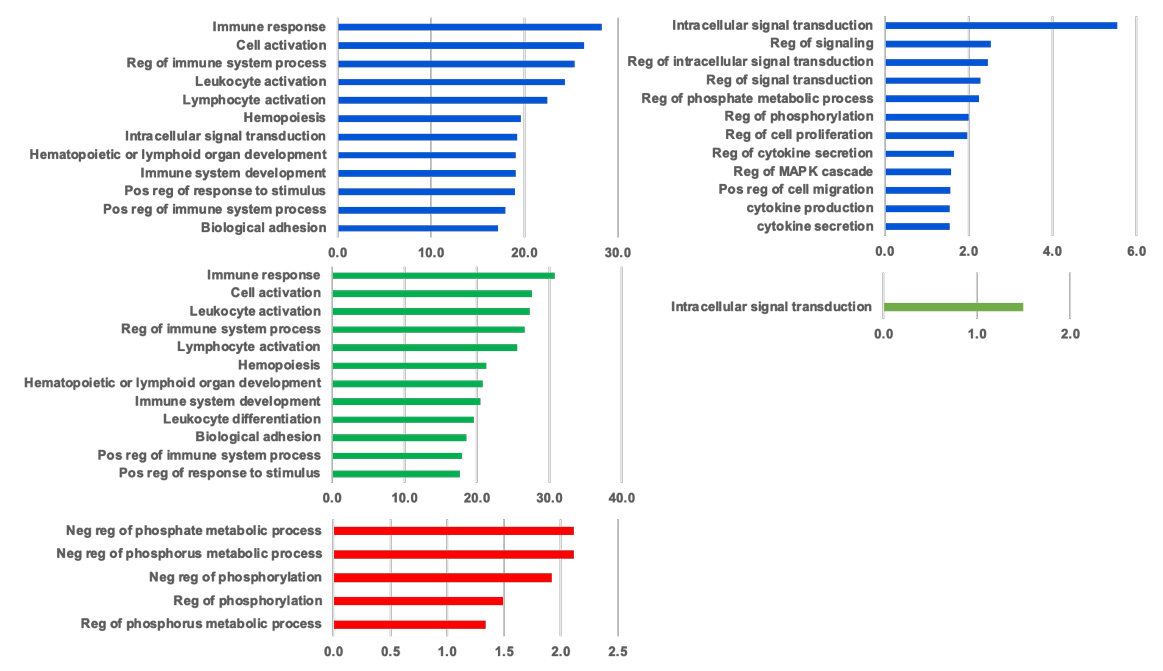


**-LOG_10_Benjamini**

**-LOG_10_Benjamini**

**All DEGs Up in Mutant Down in Mutant**
