# Supplementary material for: Differential effects of RASA3 mutations on hematopoiesis are profoundly influenced by genetic background and molecular variant
Source: PLoS Genet. 2020 Dec 28;16(12):e1008857. doi: 10.1371/journal.pgen.1008857 (PMC7793307; doi:10.1371/journal.pgen.1008857)
Supplement: S1 Table — (DOCX) [file pgen.1008857.s013.docx]

| **Group (n)** | **WBC**  **(x10^3^/µL)** | **RBC**  **(x10^6^/µL)** | **Hgb**  **(g/dL)** | **Hct**  **(%)** | **MCV**  **(fL)** | **MCH**  **(pg)** | **MCHC**  **(g/dL)** | **RDW**  **(%)** | | **HDW**  **(g/dL)** | **PLT**  **(x10^3^/µL)** | **MPV**  **(fL)** | **Retic**  **(%)** | **Spleen Weight**  **(% body wt)** | |
| --- | --- | --- | --- | --- | --- | --- | --- | --- | --- | --- | --- | --- | --- | --- | --- |
| **Control (9)** | 6.4 ± 1.6 | 10.8 ± 0.4 | 16.6 ± 0.5 | 48.7 ± 1.4 | 44.9 ± 1.4 | 15.3 ± 0.3 | 34.1 ± 0.5 | | 12.2 ± 0.6 | 1.8 ± 0.0 | 951 ± 135 | 5.7 ± 0.3 | 2.6 ± 0.4 | | 0.4 0± 0.1 |
| **Mutant (5)** | 6.1 ± 1.2 | 10.6 ± 0.7 | 16.1 ± 0.9 | 47.9 ± 2.4 | 45.1 ± 0.9 | 15.2 ± 0.3 | 33.7 ± 0.3 | | 12.3 ± 1.2 | 1.9 ± 0.0 | 750 ± 178* | 5.9 ± 0.2 | 3.7 ±1.2* | | 0.38 ± 0.3 |

All values X ± SD; WBC, white blood cell count; RBC, red blood cell count; Hgb, hemoglobin; Hct, hematocrit; MCV, mean corpuscular volume; MCH, mean corpuscular hemoglobin; MCHC, mean corpuscular hemoglobin concentration; RDW, red cell distribution width; HDW, hemoglobin distribution width; PLT, platelet count; MPV, mean platelet volume; Retic, reticulocytes. **p* < 0.05

**S1 Table. Complete blood counts in *Epor-Cre; Rasa3* adult mice 6-8 weeks of age**
