# Supplementary material for: Differential effects of RASA3 mutations on hematopoiesis are profoundly influenced by genetic background and molecular variant
Source: PLoS Genet. 2020 Dec 28;16(12):e1008857. doi: 10.1371/journal.pgen.1008857 (PMC7793307; doi:10.1371/journal.pgen.1008857)
Supplement: S2 Table — (DOCX) [file pgen.1008857.s014.docx]

**S2 Table. Complete blood counts in *scat* mice 3-5 weeks of age**

All values X ± SD; WBC, white blood cell count; RBC, red blood cell count; Hgb, hemoglobin; Hct, hematocrit; MCV, mean corpuscular volume; MCH, mean corpuscular hemoglobin; MCHC, mean corpuscular hemoglobin concentration; RDW, red cell distribution width; HDW, hemoglobin distribution width; PLT, platelet count; MPV, mean platelet volume; Retic, reticulocytes. **p* < 0.001

| **Group (n)** | **WBC**  **(x10^3^/µL)** | **RBC**  **(x10^6^/µL)** | **Hgb**  **(g/dL)** | **Hct**  **(%)** | **MCV**  **(fL)** | **MCH**  **(pg)** | **MCHC**  **(g/dL)** | **RDW**  **(%)** | **HDW**  **(g/dL)** | **PLT**  **(x10^3^/µL)** | **MPV**  **(fL)** | **Retic**  **(%)** | **Spleen Weight**  **(% body wt)** | |
| --- | --- | --- | --- | --- | --- | --- | --- | --- | --- | --- | --- | --- | --- | --- |
| **Wild type (25)** | 4.9 ± 1.2 | 8.8 ± 0.7 | 13.9 ± 1.2 | 41.6 ± 3.2 | 47.4 ± 1.3 | 15.9 ± 0.6 | 33.5 ± 1.5 | 19.7 ± 2.2 | 2.3 ± 0.2 | 595 ± 281 | 5.2 ± 0.3 | 9.5 ± 3.6 | | 0.6 ± 0.1 |
| ***scat/scat*** **(31)** | 2.8 ± 1.8* | 3.8 ± 1.1* | 5.8 ± 1.9* | 22.2 ± 6.9* | 57.8 ± 4.3* | 15.0 ± 2.2 | 25.7 ± 4.2* | 28.0 ± 6.0* | 3.3 ± 0.4* | 103 ± 106* | 8.9 ± 1.3* | 45.1 ± 12.0* | | 2.9 ± 0.6* |
