# Supplementary material for: Differential effects of RASA3 mutations on hematopoiesis are profoundly influenced by genetic background and molecular variant
Source: PLoS Genet. 2020 Dec 28;16(12):e1008857. doi: 10.1371/journal.pgen.1008857 (PMC7793307; doi:10.1371/journal.pgen.1008857)
Supplement: S3 Table — (DOCX) [file pgen.1008857.s015.docx]

**S3 Table. Complete blood counts in phlebotomized (PHB) control vs. mutant mice**

| **Group (n)** | **WBC**  **(x10^3^/µL)** | | **RBC**  **(x10^6^/µL)** | **Hgb**  **(g/dL)** | **Hct**  **(%)** | **MCV**  **(fL)** | **MCH**  **(pg)** | **MCHC**  **(g/dL)** | **RDW**  **(%)** | **HDW**  **(g/dL)** | **PLT**  **(x10^3^/µL)** | **MPV**  **(fL)** | **Retic**  **(%)** | **Spleen Weight**  **(% body wt)** | |
| --- | --- | --- | --- | --- | --- | --- | --- | --- | --- | --- | --- | --- | --- | --- | --- |
| ***Mx1-Cre*; *Rasa3***  **PHB control (6)** | | 10.1 ± 4.8 | 6.0 ± 2.7 | 9.0 ± 4.0 | 33.6 ± 15.4 | 55.3 ± 5.4 | 15.51± 1.1 | 27.3 ± 1.6 | 25.9 ± 2.8 | 3.9 ± 0.4 | 905 ± 269 | 6.1 ± 0.4 | 51.5 ± 16.8 | | 1.8 ± 0.6 |
| ***Mx1-Cre*; *Rasa3***  **mutant (6)** | | 5.7 ± 2.1 | 4.8 ± 2.4 | 7.1 ± 3.8 | 27.6 ± 13.9 | 57.7 ± 2.5 | 14.6 ± 1.5 | 25.4 ± 3.3 | 22.9 ± 6.0 | 3.6 ± 0.9 | 52 ± 67* | 12.3 ± 2.9* | 38.9 ± 17.3 | | 2.2 ± 0.8 |

All values X ± SD; WBC, white blood cell count; RBC, red blood cell count; Hgb, hemoglobin; Hct, hematocrit; MCV, mean corpuscular volume; MCH, mean corpuscular hemoglobin; MCHC, mean corpuscular hemoglobin concentration; RDW, red cell distribution width; HDW, hemoglobin distribution width; PLT, platelet count; MPV, mean platelet volume; Retic, reticulocytes. *p < 0.001
