# Supplementary material for: Differential effects of RASA3 mutations on hematopoiesis are profoundly influenced by genetic background and molecular variant
Source: PLoS Genet. 2020 Dec 28;16(12):e1008857. doi: 10.1371/journal.pgen.1008857 (PMC7793307; doi:10.1371/journal.pgen.1008857)
Supplement: S4 Table — (DOCX) [file pgen.1008857.s016.docx]

**S4 Table. Complete blood counts in B6NJ *Mx1-Cre; Rasa3* adult mice**

| **Group (n)** | **WBC**  **(x10^3^/µL)** | | **RBC**  **(x10^6^/µL)** | **Hgb**  **(g/dL)** | **Hct**  **(%)** | **MCV**  **(fL)** | **MCH**  **(pg)** | **MCHC**  **(g/dL)** | **RDW**  **(%)** | **HDW**  **(g/dL)** | **PLT**  **(x10^3^/µL)** | **MPV**  **(fL)** | **Spleen Weight**  **(% body wt)** | |
| --- | --- | --- | --- | --- | --- | --- | --- | --- | --- | --- | --- | --- | --- | --- |
| **B6NJ *Mx1-Cre; Rasa3***  **Control (3)** | | 5.7 ± 0.9 | 8.0 ± 0.3 | 12.4 ± 0.3 | 39.6 ± 0.6 | 49.8 ± 1.7 | 15.5 ± 0.5 | 31.1 ± 0.4 | 18.5 ± 1.8 | 3.0 ± 0.3 | 949 ± 260 | 7.0 ± 0.3 | | 1.1 ± 0.4 |
| **B6NJ *Mx1-Cre; Rasa3***  **mutant (3)** | | 2.7 ± 0.6* | 4.2 ± 1.0* | 6.1 ± 1.5* | 26.5 ± 4.2* | 63.5 ± 5.7^+^ | 14.4 ± 0.5 | 22.8 ± 2.3* | 21.4 ± 2.5 | 3.8 ± 0.6 | 46 ± 35* | 11.6 ± 3.1 | | 2.4 ± 0.7^+^ |

All values X ± SD; WBC, white blood cell count; RBC, red blood cell count; Hgb, hemoglobin; Hct, hematocrit; MCV, mean corpuscular volume; MCH, mean corpuscular hemoglobin; MCHC, mean corpuscular hemoglobin concentration; RDW, red cell distribution width; HDW, hemoglobin distribution width; PLT, platelet count; Retic, reticulocytes. ^+^P < 0.05, *p < 0.01
