# Supplementary material for: Differential effects of RASA3 mutations on hematopoiesis are profoundly influenced by genetic background and molecular variant
Source: PLoS Genet. 2020 Dec 28;16(12):e1008857. doi: 10.1371/journal.pgen.1008857 (PMC7793307; doi:10.1371/journal.pgen.1008857)
Supplement: S5 Table — (DOCX) [file pgen.1008857.s017.docx]

**S5 Table. Complete blood counts in C57BL/6J-*hlb381* mice 3-4 weeks of age**

| **Genotype (n)** | **WBC**  **(x10^3^/µL)** | **RBC**  **(x10^6^/µL)** | **Hgb**  **(g/dL)** | **Hct**  **(%)** | **MCV**  **(fL)** | **MCH**  **(pg)** | **MCHC**  **(g/dL)** | **RDW**  **(%)** | | **HDW**  **g/dL)** | **PLT**  **(x10^3^/µL)** | **MPV**  **(fL)** | | **Retic**  **(%)** | |  |
| --- | --- | --- | --- | --- | --- | --- | --- | --- | --- | --- | --- | --- | --- | --- | --- | --- |
| **Females** | | | | | | | | | | | | | | | | |
| ***+/+* (5)** | 6.7 ± 1.3 | 8.6 ± 0.6 | 13.3 ± 0.6 | 44.6 ± 3.1 | 52.0 ± 0.7 | 15.6 ± 0.4 | 30.0 ± 0.9 | | 17.6 ± 1.5 | 2.0 ± 0.1 | 925 ± 148 | | 6.8 ± 0.5 | | 11.4 ± 3.8 | |
| ***hlb381/+* (14)** | 5.9 ± 1.5 | 8.7 ± 0.7 | 12.7 ± 1.4 | 44.3 ± 3.7 | 50.8 ± 1.1 | 14.5 ± 1.0^ | 28.6 ± 1.7 | | 19.6 ± 6.1 | 2.1 ± 0.5 | 1015 ± 193 | | 7.3 ± 0.8 | | 14.8 ± 11.7 | |
| ***hlb381/hlb381* (13)** | 2.4 ± 1.1*^+^ | 8.6 ± 0.9 | 12.2 ± 1.3 | 42.7 ± 3.3 | 50.1 ± 2.3 | 14.3 ± 0.7^ | 28.6 ± 1.3 | | 18.7 ± 3.6 | 2.4 ± 0.5 | 29 ± 12*^+^ | | 8.1 ± 1.0^^#^ | | 18.3 ± 8.6 | |
| **Males** | | | | | | | | | | | | | | | | |
| ***+/+* (9)** | 5.4 ± 1.4 | 8.5 ± 0.5 | 13.0 ± 0.6 | 43.7 ± 2.8 | 51.1 ± 0.6 | 15.2 ± 0.4 | 29.6 ± 0.9 | | 17.3 ± 1.4 | 1.9 ± 0.1 | 983 ± 164 | | 6.9 ± 0.6 | | 11.1 ± 4.1 | |
| ***hlb381/+* (16)** | 6.2 ± 0.9 | 8.9 ± 0.4 | 13.0 ± 0.7 | 44.6 ± 2.2 | 50.2 ± 1.1 | 14.6 ± 0.5^ | 29.1 ± 0.8 | | 16.9 ± 2.3 | 1.8 ± 0.2 | 1020 ± 213 | | 6.8 ± 0.5 | | 7.0 ± 1.1 | |
| ***hlb381/hlb381* (15)** | 2.1 ± 0.8*^+^ | 8.6 ± 0.5 | 12.2 ± 0.7^^+^ | 42.5 ± 2.5 | 49.6 ± 1.8^ | 14.2 ± 0.5* | 28.7 ± 1.2^ | | 18.8 ± 2.8 | 2.3 ± 0.4* | 25 ± 5*^+^ | | 7.7 ± 0.9^^+^ | | 18.3 ± 8.0*^+^ | |

| **Genotype (n)** | **Spleen Weight**  **(% BW)** | |
| --- | --- | --- |
| ***+/+* (15)** | | 0.6 ± 0.2 |
| ***hlb381/+* (8)** | | 0.6 ± 0.1 |
| ***hlb381/hlb381* (22)** | | 0.9 ± 0.3*^+^ |

Number in parentheses = sample n. All values X ± SD; *p < 0.01 *vs*. +/+; ^p < 0.05 *vs*. +/+; ^+^p < 0.01 *vs.* *hlb381*/+; ^#^p < 0.05 *vs*. *hlb381*/+

WBC, white blood cell count; RBC, red blood cell count; Hgb, hemoglobin; Hct, hematocrit; MCV, mean corpuscular volume; MCH, mean corpuscular hemoglobin; MCHC, mean corpuscular hemoglobin concentration; RDW, red cell distribution width; HDW, hemoglobin distribution width; PLT, platelet count; MPV, mean platelet volume; Retic, reticulocytes; BW, body weight

**Spleen weights in *hlb381* mice**

**3-4 weeks of age (males and**

**females combined)**
