# Supplementary material for: Differential effects of RASA3 mutations on hematopoiesis are profoundly influenced by genetic background and molecular variant
Source: PLoS Genet. 2020 Dec 28;16(12):e1008857. doi: 10.1371/journal.pgen.1008857 (PMC7793307; doi:10.1371/journal.pgen.1008857)
Supplement: S6 Table — (DOCX) [file pgen.1008857.s018.docx]

**S6 Table. Complete blood counts in C57BL/6J-*hlb381* mice ≥ 6 weeks of age**

| **Genotype** | **WBC**  **(x10^3^/µL)** | **RBC**  **(x10^6^/µL)** | **Hgb**  **(g/dL)** | **Hct**  **(%)** | **MCV**  **(fL)** | **MCH**  **(pg)** | **MCHC**  **(g/dL)** | **RDW**  **(%)** | **HDW**  **(g/dL)** | **PLT**  **(x10^3^/µL)** | **MPV**  **(fL)** | **Retic**  **(%)** |  | |  |
| --- | --- | --- | --- | --- | --- | --- | --- | --- | --- | --- | --- | --- | --- | --- | --- |
| **Females** | | | | | | | | | | | | | |  |  |
| **B6-+/+ (7)** | 9.5 ± 1.3 | 10.4 ± 0.2 | 15.5 ± 0.3 | 48.4 ± 0.9 | 46.8 ± 0.3 | 15.0 ± 0.2 | 32.1 ± 0.2 | 13.0 ± 0.3 | 1.8 ± 0.1 | 972 ± 146 | 4.9 ± 0.2 | 3.1 ± 0.6 | |  | |
| ***hlb381*/+ (8)** | 8.0 ± 1.0^ | 10.5 ± 0.6 | 15.6 ± 0.8 | 50.3 ± 2.9 | 47.8 ± 1.8 | 14.8 ± 0.2 | 31.1 ± 1.1^+^ | 12.8 ± 0.8 | 1.7 ± 0.1 | 1116 ± 23^ | 6.7 ± 0.7 | 3.8 ± 0.5 | |  | |
| ***hlb381/381* (16)** | 3.4 ± 0.5* | 10.2 ± 0.4 | 15.1 ± 0.5 | 48.7 ± 1.6 | 47.7 ± 1.1 | 14.8 ± 0.2 | 31.0 ± 0.6^ | 13.0 ± 0.5 | 1.9 ± 0.1 | 28 ± 15* | 8.8 ± 2.0* | 6.3 ± 1.0* | |  | |
| **Males** | | | | | | | | | | | | | |  |  |
| **B6-+/+ (20)** | 9.4 ± 1.7 | 10.4 ± 0.3 | 15.3 ± 0.3 | 48.8 ± 1.2 | 47.1 ± 0.5 | 14.8 ± 0.2 | 31.4 ± 0.4 | 12.5 ± 0.5 | 1.8 ± 0.0 | 1061 ± 93 | 4.8 ± 0.2 | 3.9 ± 0.4 | |  | |
| ***hlb381*/+ (12)** | 7.6 ± 0.9^ | 9.7 ± 1.5 | 14.3 ± 2.2 | 47.6 ± 8.1 | 48.8 ± 2.2^+^ | 14.7 ± 0.2 | 30.2 ± 1.1* | 13.4 ± 1.4^+^ | 1.7 ± 0.2 | 1114 ± 161 | 6.5 ± 0.4* | 4.9 ± 2.6 | |  | |
| ***hlb381/381* (12)** | 3.8 ± 0.6* | 9.7 ± 00.8 | 14.0 ± 0.8^+^ | 47.6 ± 2.1 | 48.4 ± 2.1 | 14.5 ± 0.4^+^ | 29.9 ± 0.7* | 13.7 ± 0.7^ | 1.9 ± 0.1^+^ | 30 ± 18* | 8.9 ± 2.0* | 8.2 ± 3.1* | |  | |

Number in parentheses = sample n. All values X ± SD. * p < 0.001,

^ p < 0.01, ^+^ p < 0.05 *vs* control (+/+) mice.

WBC, white blood cell count; RBC, red blood cell count; Hgb, hemoglobin; Hct, hematocrit; MCV, mean corpuscular volume; MCH, mean corpuscular hemoglobin; MCHC, mean corpuscular hemoglobin concentration; RDW, red cell distribution width; HDW, hemoglobin distribution width; PLT, platelet count; MPV, mean platelet volume; Retic, reticulocytes; BW, body weight

**Spleen weights in *hlb381* mice**

**≥ 6 weeks of age (males and**

**females combined)**

| **Genotype (n)** | **Spleen Weight**  **(% BW)** | |
| --- | --- | --- |
| ***+/+* (4)** | | 0.3 ± 0.0 |
| ***hlb381/hlb381* (3)** | | 0.5 ± 0.1^ |
