# Supplementary material for: Differential effects of RASA3 mutations on hematopoiesis are profoundly influenced by genetic background and molecular variant
Source: PLoS Genet. 2020 Dec 28;16(12):e1008857. doi: 10.1371/journal.pgen.1008857 (PMC7793307; doi:10.1371/journal.pgen.1008857)
Supplement: S7 Table — (DOCX) [file pgen.1008857.s019.docx]

**S7 Table. RNAseq biological replicates**

| Tissue → | Bone Marrow | | | Spleen | | |
| --- | --- | --- | --- | --- | --- | --- |
| Group ↓ | **Whole** | **MEP** | **SMP** | **Whole** | **MEP** | **SMP** |
| WT littermate | 3 | 5 | 5 | 3 | 4 | 4 |
| *scat* crisis | 3 | 3 | 3 | 3 | 3 | 3 |
| *scat* partial remission | 3 | 3 | 3 | 3 | 3 | 3 |
