# Supplementary material for: Differential effects of RASA3 mutations on hematopoiesis are profoundly influenced by genetic background and molecular variant
Source: PLoS Genet. 2020 Dec 28;16(12):e1008857. doi: 10.1371/journal.pgen.1008857 (PMC7793307; doi:10.1371/journal.pgen.1008857)
Supplement: S8 Table — (DOCX) [file pgen.1008857.s020.docx]

**S8 Table. Complete blood counts in *scat* mice used for RNAseq analyses**

| **Group (n)** | **WBC**  **(x10^3^/µL)** | **RBC**  **(x10^6^/µL)** | **Hgb**  **(g/dL)** | **Hct**  **(%)** | **MCV**  **(fL)** | **MCH**  **(pg)** | **MCHC**  **(g/dL)** | **RDW**  **(%)** | **HDW**  **(g/dL)** | **PLT**  **(x10^3^/µL)** | **MPV**  **(fL)** | **Retic**  **(%)** | **Spleen Weight**  **(% body wt)** | |
| --- | --- | --- | --- | --- | --- | --- | --- | --- | --- | --- | --- | --- | --- | --- |
| **WT** | 6.0 ± 0.5 | 8.9 ± 1.1 | 14.0 ± 1.5 | 42.1± 4.0 | 47.6 ± 1.2 | 15.9 ± 0.5 | 33.3 ± 0.9 | 18.2 ± 2.6 | 2.2 ± 0.1 | 716 ± 341 | 5.0 ± 0.1 | 13.1 ± 6.0 | | 0.6 ± 0.1 |
| ***scat* crisis** | 2.9 ± 2.1 | 3.0 ± 0.1 | 4.5 ± 1.0 | 16.2 ± 3.0 | 54.0 ± 7.6 | 15.2 ± 3.6 | 28.9 ± 10.3 | 36.7 ± 3.0 | 3.6 ± 0.6 | 266 ± 90 | 8.9 ± 0.5 | 78.9 ± 14.2 | | 3.1 ± 0.9 |
| ***scat* partial remission** | 2.7 ± 0.6 | 4.9 ± 0.5 | 7.8 ± 0.8 | 28.3 ± 2.1 | 57.6 ± 2.3 | 15.9 ± 0.5 | 27.5 ± 1.3 | 23.3 ± 2.7 | 2.9 ± 0.4 | 28 ± 13 | 8.1 ± 1.0 | 47.7 ± 16.0 | | 2.5 ± 0.5 |

All values X ± SD; WBC, white blood cell count; RBC, red blood cell count; Hgb, hemoglobin; Hct, hematocrit; MCV, mean corpuscular volume; MCH, mean corpuscular hemoglobin; MCHC, mean corpuscular hemoglobin concentration; RDW, red cell distribution width; HDW, hemoglobin distribution width; PLT, platelet count; Retic, reticulocytes.
