# Supplementary material for: Differential effects of RASA3 mutations on hematopoiesis are profoundly influenced by genetic background and molecular variant
Source: PLoS Genet. 2020 Dec 28;16(12):e1008857. doi: 10.1371/journal.pgen.1008857 (PMC7793307; doi:10.1371/journal.pgen.1008857)
Supplement: S9 Table — (DOCX) [file pgen.1008857.s021.docx]

**S9 Table. Mouse strains**

| **Strain name** | **JAX* Stock No** | **Description, use** | **Abbreviation** |
| --- | --- | --- | --- |
| C57BL/6J | 000664 | Inbred strain | B6J |
| C57BL/6NJ | 005304 | Inbred strain | B6NJ |
| 129S1/SvImJ | 002448 | Inbred strain | 129S1 |
| CbyJ.Cby-*Rasa3^scat^/*Llp | 012911 | Mutant strain | *scat* |
| B6.CBy-*Rasa3^scat^/*Llp | – | Mutant strain | *scat* |
| C57BL/6J- *Rasa3^hlb381^/J* | 005343 | Mutant strain | *hlb381* |
| C57BL/6N-*Rasa3^tm1a(KOMP)J^*/J | 021992 | KOMP conditional ready model | *–* |
| B6J;129S1-*Rasa3^tm1Llp^*/Llp | – | Peters cKO Model | – |
| B6.Cg-Tg(ACTFLPe)9205Dym/J | 005703 | *FLP1* recombinase | *ACT-Flpe* |
| BALB/cJ-*Epor^tm1(EGFP/cre)Uk^* | – | Cre-expressing strain | *Epor-Cre* |
| B6.Cg-Tg(Mx1-cre)1Cgn/J | 003556 | Cre-expressing strain | *Mx1-Cre* |
| B6.C-Tg(CMV-cre)1Cgn/J | 006054 | Cre-expressing strain | *CMV-Cre* |
| B6.Cg-Tg(Tek-cre)1Ywa/J | 008863 | Cre-expressing strain | *Tie2-Cre* |
| B6.Cg-*Commd10^Tg(Vav1-icre)A2Kio^/*J | 008610 | Cre-expressing strain | *Vav-Cre* |
| FVB-Tg(Csf1r-icre)1Jwp/J | 021024 | Cre-expressing strain | *Csf1r-Cre* |
| B6N.Cg-*Edil3^Tg(Sox2-cre)1Amc^*/J | 014094 | Cre-expressing strain | *Sox2-Cre* |
