# Supplementary material for: Differential effects of RASA3 mutations on hematopoiesis are profoundly influenced by genetic background and molecular variant
Source: PLoS Genet. 2020 Dec 28;16(12):e1008857. doi: 10.1371/journal.pgen.1008857 (PMC7793307; doi:10.1371/journal.pgen.1008857)
Supplement: S10 Table — (DOCX) [file pgen.1008857.s022.docx]

| **Primers** | **Forward** | **Reverse** | **Product detected (bp)** |
| --- | --- | --- | --- |
| 5’ probe | TCTGCAGTACATGGAGTTCCA | TCCCTCCCTACTCACAGTTC | 5’ probe for Southern blot (738) |
| 3’ probe | AAAACTCCCTGTCGGGCAA | AAAAACCCCACCCCAAGA | 3’ probe for Southern blot (649) |
| NeoGT-F | GATCTCATGCTGGAGTTCTTCGCC | Rasa3GT-R | cKO allele (1300) |
| Rasa3GT-F/R | TCCTCTTCCTAGTGTCGTGAGCAA | TCCAGTGTTACGGTCGTCTGCATA | Following Flpe-mediated excision:  WT allele (940)  floxed allele (1109)  null (ΔEx3) allele (315) |
| Rasa3 Exons 1-7 | CTTCCAGAGCGTGAGGATCAAGATT | ATAAAACACCTCGTCAAACTGGGGG | 568 |
| Rasa3 Exons 7-12 | CCCCCAGTTTGACGAGGTGTTTTAT | TCAATGGTGGGCTTCAGGGTCACAT | 630 |
| Rasa3 Exons 12-19 | ATGTGACCCTGAAGCCCACCATTGA | GCTTTTCTGGTAGGTGAACTCGTGG | 668 |
| Rasa3 Exons 19-24 | CCACGAGTTCACCTACCAGAAAAGC | TTAAATGGAATGGGTGGAGATCTCA | 685 |

**S10 Table. Genotyping primers and product sizes**
