# Supplementary material for: Differential effects of RASA3 mutations on hematopoiesis are profoundly influenced by genetic background and molecular variant
Source: PLoS Genet. 2020 Dec 28;16(12):e1008857. doi: 10.1371/journal.pgen.1008857 (PMC7793307; doi:10.1371/journal.pgen.1008857)
Supplement: S11 Table — (DOCX) [file pgen.1008857.s023.docx]

| **S11 Table. Flow cytometry antibodies** | | | | |
| --- | --- | --- | --- | --- |
| **Purpose** | **Antibody** | **Conjugate** | **Clone** | **Source** |
| **Lineage depletion cocktail** | CD3e | Biotin | 145-2C11 | BDBiosciences |
|  | CD4 | Biotin | GK1.5 | BioLegend |
|  | CD8 | Biotin | 53-6.72 | BDBiosciences |
|  | CD11b | Biotin | M1/70 | eBioSciences |
|  | CD19 | Biotin | 1D3 | BDBiosciences |
|  | CD161 | Biotin | PK136 | eBioSciences |
|  | Ly6G/C | Biotin | RB6-8C5 | eBioSciences |
|  | Ter119 | Biotin | Ter119 | eBioSciences |
| **Erythroid precursors** | Ter119 | PE |  | BioLegend |
|  | CD45 | PE-Cy7 | 30-F11 | BDBiosciences |
|  | CD44 | APC | IM7.8.1 | Tonbo Biosciences |
|  | Ly6G/C | Biotin | RB6-8C5 | eBioSciences |
|  | CD11b | Biotin | M1/70 | eBioSciences |
| **qRT-PCR** | Lineage depletion cocktail |  |  |  |
|  | Sca-1 | PE-Cy5 | D7 | BioLegend |
|  | CD117 | PE-Cy7 | 2B8 | eBioSciences |
|  | CD34 | APC | RAM34 | BioLegend |
|  | CD16/32 | BV421 | 93 | BioLegend |
|  | CD41 | FITC | MWReg30 | eBioSciences |
|  | CD135 | PE | A2F10 | BioLegend |
| **RNAseq (SMP, MEP)** | Lineage depletion cocktail |  |  |  |
|  | CD117 | APC | 2B8 | BDBiosciences |
|  | CD34 | Fitc | RAM34 | BDBiosciences |
|  | CD16/32 | BV421 | 93 | BioLegend |
|  | Sca-1 | PE-Cy5 | D7 | BioLegend |
| **Hematopoietic Stem/Progenitors** | Lineage depletion cocktail |  |  |  |
|  | CD34 | FITC | RAM34 | BDBiosciences |
|  | CD150/SLAM | PerCP-Cy5.5 | TC15-12F12.2 | BioLegend |
|  | CD135 | PE | A2F10 | eBioSciences |
|  | CD127 | PE-CF594 | A7R34 | BDBiosciences |
|  | Sca-1 | PE-Cy5 | D7 | BioLegend |
|  | CD117 | APC | 2B8 | BDBiosciences |
|  | CD16/32 | e450 | 93 | eBioSciences |
